# Quantifying Motor Task Performance by Bounded Rational Decision Theory

**DOI:** 10.3389/fnins.2018.00932

**Published:** 2018-12-14

**Authors:** Sonja Schach, Sebastian Gottwald, Daniel A. Braun

**Affiliations:** Faculty of Engingeering, Computer Science and Psychology, Institute of Neural Information Processing, Ulm University, Ulm, Germany

**Keywords:** bounded rationality, motor control, movement planning, optimality model, reaction time, information-processing resources, computational cost

## Abstract

Expected utility models are often used as a normative baseline for human performance in motor tasks. However, this baseline ignores computational costs that are incurred when searching for the optimal strategy. In contrast, bounded rational decision-theory provides a normative baseline that takes computational effort into account, as it describes optimal behavior of an agent with limited information-processing capacity to change a prior motor strategy (before information-processing) into a posterior strategy (after information-processing). Here, we devised a pointing task where subjects had restricted reaction and movement time. In particular, we manipulated the permissible reaction time as a proxy for the amount of computation allowed for planning the movements. Moreover, we tested three different distributions over the target locations to induce different prior strategies that would influence the amount of required information-processing. We found that movement endpoint precision generally decreases with limited planning time and that non-uniform prior probabilities allow for more precise movements toward high-probability targets. Considering these constraints in a bounded rational decision model, we found that subjects were generally close to bounded optimal. We conclude that bounded rational decision theory may be a promising normative framework to analyze human sensorimotor performance.

## 1. Introduction

According to ethological theory (Alcock, 2001), behavior of living beings can be explained following two kinds of arguments. In proximate explanations, behavior results from a change in physiological and environmental states that are caused by natural laws of physics and biochemistry. Such mechanistic explanations typically answer the “how”-question of behavior. In contrast, in ultimate explanations we ask “why”-questions that are typically answered in an evolutionary context elucidating the usefulness of the behavior and the adaptive advantage it might bring to the organism. A bridge between the two kinds of explanations may be provided by optimal actor models (Parker and Maynard Smith, 1990; Todorov, 2004). Abstractly, an optimal actor model reproduces an observed behavior by minimizing (or maximizing) a cost (or reward) function that depends on certain critical variables and some constraints on these variables (e.g., environmental dynamics). Naturally, such optimal actor models fit an ultimate explanation of behavior by emphasizing usefulness, suggesting an evolutionary relevance of the critical variables considered. However, by considering the nature of the variables also some light may be shed on the “how”-question, as these variables may be critical for information-processing especially during learning, without specifying a detailed mechanism.

In human motor control, for example, a wide range of behaviors has been previously explained by optimal actor models that depend on critical variables such as motor effort, trajectory smoothness, endpoint variability, task accuracy, or endstate comfort (Flash and Hogan, 1985; Kawato et al., 1990; Harris and Wolpert, 1998; Trommershäuser et al., 2003a,b, 2006, 2008; Todorov, 2004). As motor control is fundamentally affected by uncertainty that arises both from our own bodies and from the environment (Faisal et al., 2008), optimal actor models are typically expressed as Bayesian optimal actor models that deal efficiently with uncertainty by averaging over all possibilities and optimizing expected costs (or rewards). Bayesian models have been very successful in explaining human perceptual and sensorimotor learning by providing evidence that the sensorimotor system integrates prior knowledge with new incoming information to make inferences about unobserved latent variables in a way that is consistent with Bayesian statistics (van Beers et al., 1999; Ernst and Banks, 2002; Knill and Pouget, 2004; Körding and Wolpert, 2004, 2006; Todorov, 2004; Hudson et al., 2007; Körding, 2007; Wolpert, 2007; Braun et al., 2009; Girshick and Banks, 2009; Wu et al., 2009; Turnham et al., 2011; Wolpert and Landy, 2012). This research has culminated in the Bayesian brain hypothesis that stipulates that the brain is constantly updating predictions about its environment consistent with Bayesian probabilities and using these probabilities for optimal acting (Doya et al., 2007).

While Bayesian models are conceptually appealing, we know in particular from research in artificial intelligence that such models can become wildly intractable when faced with real-world information-processing problems. A growing number of studies has therefore addressed the question under what conditions we observe deviations from Bayes-optimal behavior. Acerbi et al. (2014), for example, have investigated suboptimality in probabilistic inference in a pointing task and found that the degree of suboptimality was dependend on the shape of the priors. In rapid pointing tasks human performance is reported to deviate from optimality in configurations with markedly asymmetric expected gain landscapes (Wu et al., 2006). Similar deviations from expected gain maximization in the presence of spatial asymmetry have also been reported for whole-body movements (O'Brien and Ahmed, 2013). While these studies have used different ways to quantify deviations from Bayes-optimal behavior, there is no agreed-upon framework to study such deviations.

Here, we propose information-theoretic bounded rationality (Ortega and Braun, 2011, 2013; Genewein et al., 2015) as a unified framework that encompasses a wide range of previous information-theoretic models of perception-action systems (McKelvey and Palfrey, 1995; Mattsson and Weibull, 2002; Sims, 2003; Wolpert, 2004; Still, 2009; Todorov, 2009; Friston, 2010; Tishby and Polani, 2011; Kappen et al., 2012) to study the efficiency of optimal actors with limited information-processing capabilities. In such models the information-processing cost is measured as the relative Shannon information between a prior distribution (before information-processing) and a posterior distribution (after information-processing). This change in Shannon information provides an abstract measure that can be monotonically mapped onto any resource cost (e.g., time or effort), as any sensible expenditure on resources should aim to increase the ability to differentiate between different options. Here, we apply this framework to a motor control task involving fast reaching movements where we manipulate both the reaction time as a proxy for the permissible amount of information-processing and prior probabilities of motor strategies that would influence the amount of relative Shannon information generated during information-processing. We demonstrate how to use this framework to quantify subjects' efficiency taking into account their information-processing costs.

## 2. Methods

### 2.1. Theoretical Methods

#### Bounded Rationality Model

In our experiment we consider a decision-maker that is confronted with a world state w∈W and chooses an action a∈A which may lead to the consequence x∈X with known probability *p*(*x*|*a*). The decision-maker's preferences are represented by a utility function *U*(*w, x*). A perfectly rational decision-maker would choose their action according to

(1) $$\begin{array}{r} a^{*}(w)=\arg\underset{a}{\max}E_{p(x|a)}[U(w,x)]=\arg\underset{a}{\max}V(w,a), \end{array}$$

where we have introduced *V*(*w, a*): = 𝔼_*p*(*x*|*a*)_[*U*(*w, x*)] to represent the utility of choosing action *a* in context *w*. In the following we assume that the decision-maker knows the utility function in the sense that *V*(*w, a*) can be queried for different instances *w* and *a*, and that the maximum utility can be found by spending resources in a search process. The basic idea of bounded rational decision-making can be best illustrated in the simplest scenario (Ortega and Braun, 2011, 2013; Genewein et al., 2015), when there is only one world state that the decision-maker has to adapt to. The decision-maker then optimizes the utility function by querying *V*(*a*) during a deliberation phase before selecting the action *a*. Assuming the actor has a prior decision strategy *p*_0_(*a*) means that in case of no available information-processing resources the actor chooses *a* by sampling from *p*_0_(*a*). During deliberation the bounded rational actor searches for high-utility options and the actor's strategy changes from *p*_0_(*a*) to p^*^(*a*) where

(2) $$\begin{array}{r} p^{*}(a)=\arg\underset{p(a)}{\max}E_{p(a)}[V(a)]\text{ s.t. }D_{KL}(p(a)\|p_{0}(a))\leq C. \end{array}$$

The Kullback-Leibler divergence *D*_*KL*_(*p*(*a*)||*p*_0_(*a*)) is the relative entropy between the distributions *p*_0_(*a*) and *p*(*a*) and measures the amount of Shannon information available to the actor. The more resources (e.g., time, money, or amount of samples) available to the actor, the higher the amount of Shannon information *C* that the actor commands, i.e., the better the actor can discriminate between the different options. Effectively, the actor faces a trade-off between information-processing costs and the expected gain in utility.

In the case of multiple world states *w*, the utility *V*(*w, a*) depends on *w* and *a*. If we want to formalize information-processing of the stimulus, we choose a prior strategy *p*_0_(*a*) that only depends on *a* and a posterior strategy *p*(*a*|*w*) resulting from a deliberation phase that is triggered by a change in world state. Assuming a known world state distribution ρ(*w*), the bounded rational posterior will now be given by

(3) $$\begin{array}{r} \begin{array}{l} p^{*}(a|w)=\arg\underset{p(a|w)}{\max}E_{\rho (w)}\left[E_{p(a|w)}[V(a,w)]\right]\\ \text{ s.t. }E_{\rho (w)}\left[D_{KL}(p(a|w)\|p_{0}(a))\right]\leq C. \end{array} \end{array}$$

Here, the average *D*_*KL*_ measures the average distance between the prior *p*_0_(*a*) and all possible posteriors *p*(*a*|*w*) weighted by the probability of their occurrence ρ(*w*). This allows for the consideration of the optimal prior p^*^(*a*) that has the minimum average information distance to all the posteriors *p*(*a*|*w*) of the different world states *w*, that is the prior that would allow for resource optimal processing for each *w*. In this case, the bounded rational decision-making problem can be written as

(4) $$\begin{array}{r} \begin{array}{l} p^{*}(a|w)=\arg\underset{p(a|w)}{\max}E[V(a,w)]\\ \text{ s.t. }E\left[D_{KL}(p(a|w)\|p^{*}(a))\right]\leq C, \end{array} \end{array}$$

where $p^{*}\left(a\right)=\underset{w}{\sum }\rho \left(w\right)p\left(a|w\right)$ is the optimal prior given by the marginal, and expectations 𝔼[·] are taken as in Equation 3. Assuming a Lagrange multiplier denoted by β, the constrained optimization problem in Equation 4 can be equivalently rewritten as

(5) $$\begin{array}{r} p^{*}(a|w)=\arg\underset{p(a|w)}{\max}\left(E[V(w,a)]-\frac{1}{\beta }I(W;A)\right), \end{array}$$

with the mutual information $I\left(W;A\right)=E\left[D_{KL}\left(p\left(a|w\right)||p^{*}\left(a\right)\right)\right]$ and the solution equations

(6) $$\begin{array}{r} \{\begin{array}{l} p^{*}(a|w)=\frac{1}{Z(w)}p^{*}(a)e^{\beta V(w,a)}\\ p^{*}(a)=\sum_{w}\rho (w)p^{*}(a|w) \end{array} \end{array}$$

with *Z*(*w*): = $\underset{A}{\int }p^{*}\left(a\right)e^{\beta V\left(a,w\right)}da$ (see Supplementary Material 1 for a detailed derivation). This problem formulation is equivalent to the rate distortion problem in information theory and can be solved by the Blahut-Arimoto algorithm. The parameter β is determined by the available information resources *C*. In the limit β → ∞ we obtain the perfectly rational decision-maker with unlimited resources. In the limit β → 0 we obtain the resourceless decision-maker that has to act according to its prior.

**Figure 1:**
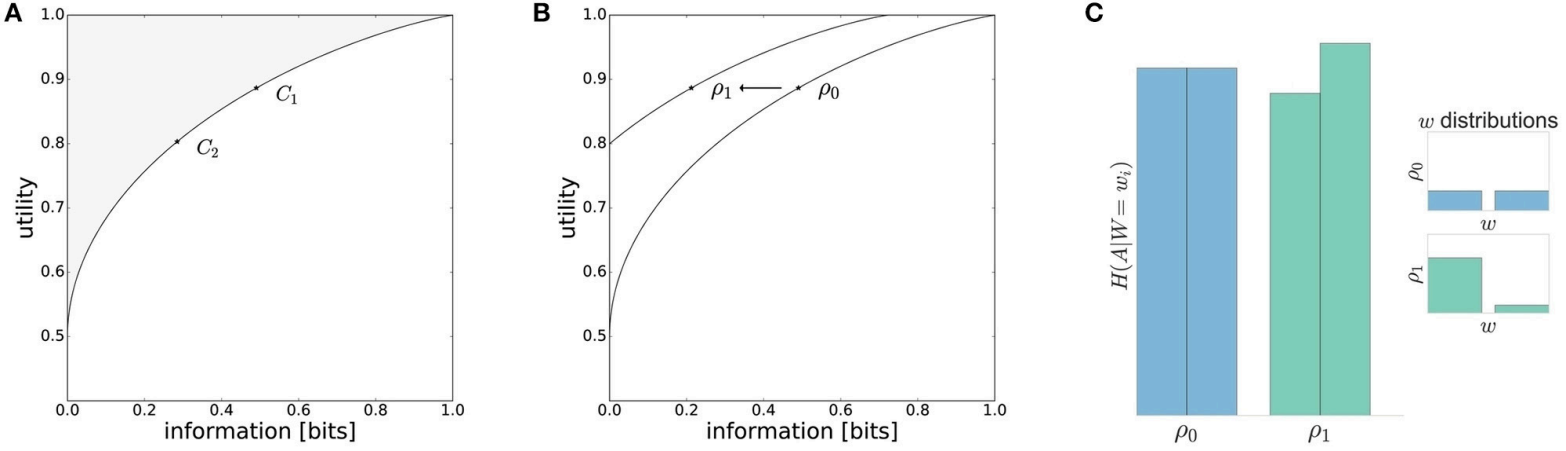
Model predictions. **(A)** The performance of a decision-maker depends on the resource available. With high available resource *C*_1_ the information processing cost is higher and higher utility is achieved compared to the low resource scenario *C*_2_. **(B)** The information-processing cost measured in bits is reduced for movement decisions under a low-entropy world-state distribution ρ_1_(*w*). In the case of perfect adaptation, there will be a different efficiency frontier when changing the world state distribution. **(C)** For different world state distributions the entropy of action distributions *H*(*A*|*W* = *w*_*i*_) changes. A higher world state probability lowers the entropy whereas low world state probabilities are associated with higher action entropies.

By varying the parameter β from 0 to ∞, we obtain a family of bounded rational solutions p^*^(*a*|*w*), such that for each β we can compute the corresponding information resource *I*(*W*; *A*) and the corresponding performance 𝔼[*V*(*w, a*)]. By exploiting the known relationship *p*(*x*|*a*), the family of solutions can also be represented in the space of consequences *x* by

(7) $$\begin{array}{r} p^{*}(x|w)=\int_{A}p(x|a)p^{*}(a|w)da, \end{array}$$

with performance criterion $E\left[U\left(w,x\right)\right]=E_{\rho \left(w\right)}E_{p^{*}\left(x|w\right)}\left[U\left(w,x\right)\right]$ and the effective resource *I*(*W*; *X*). These optimal solutions form an efficiency frontier that can be illustrated pictorially in a two-dimensional plot, where the abscissa denotes the resources spent between prior and posterior measured in units of information bits and where the ordinate shows the utility achieved by the decision-maker. The bounded rational solutions form a continuous curve of Pareto optima, i.e., either of the two quantities can only be improved by making the other one worse (see Figure 1A).

The bounded rational decision-making model makes the following predictions:

- Any decision-maker, irrespective of the underlying mechanism, can either be on or below the curve, i.e., given a certain amount of information resources it is not possible to achieve a higher expected utility than the bounded rational actor.
- If we vary the resources available to the decision-making process (e.g., *C*_1_ and *C*_2_ in Figure 1A) and measure both the utility achieved and the information produced, then we expect more resources to be associated with higher information-processing costs and higher utility. Note, this prediction is compatible with most mechanistic decision-making models, but follows here from normative information-theoretic resource considerations.
- If we vary the world state distribution ρ(*w*) by moving from a high-entropy (e.g., uniform) to a low-entropy (e.g., strongly non-uniform) distribution, then by adapting prior and posterior the decision-maker can lower information processing costs without compromising the average performance [e.g., ρ_0_(*w*) and ρ_1_(*w*) in Figure 1B]. In particular, one would expect that high-probability world-states should be associated with low-entropy action distributions, and low-probability world-states with high-entropy action distributions (see Figure 1C).

#### Measuring Bounded Rational Decision-Makers

If we want to compare real-world decision-makers with the above theoretical description, we need to determine two quantities experimentally, namely the average utility *U*^*exp*^ and the average empirical information $D_{KL}^{exp}$. From the experiment we typically obtain world-state dependent choice probabilities, from which it is straightforward to determine average performance *U*^*exp*^. The choice probabilities are the measured posteriors that may differ from the theoretical bounded optimal posteriors. In addition to the posteriors, we need to determine the prior choice probabilities in order to obtain $D_{KL}^{exp}$. In principle, the prior choice probabilities could be determined by interspersing choice trials with close to zero resources (*β* close to zero), if we assume that adaptation of the prior happens much more slowly than the decision-making process itself. However, in practice reducing resources to zero may prove difficult, as this may lead to particular no-response behavior instead of random behavior sampled from the prior. Similar to the posterior choice probabilities, the prior choice probabilities can be optimal or suboptimal depending on whether adaptation to the task was complete or incomplete. If the decision-maker does not adapt at all, this corresponds to a fixed prior *p*_0_(*a*). If the decision-maker knows the distribution ρ(*w*) over world states and adapts its prior perfectly, the prior will be given by the marginal *p*(*a*) from Equation (6). In general, the prior can be anywhere in-between these two extremes. Given both priors and posteriors, we can then determine the average empirical information $D_{KL}^{exp}$ that is generated by the decision-making process. In case of perfect adaptation the average empirical information is equal to the mutual information $D_{KL}^{exp}=I^{exp}$, i.e., the information can be measured with respect to the actual statistics of the marginal rather than with respect to a sub-optimal subjective prior.

The performance of the decision-maker can be represented by a data point $\left(U^{exp},D_{KL}^{exp}\right)$ in the utility-information-plane. Given $\left(U^{exp},D_{KL}^{exp}\right)$, we can compute the efficiency of a decision-maker by determining

(8) $$\begin{array}{r} \epsilon =\frac{U^{\exp}-U^{\min}}{U^{\max}-U^{\min}}, \end{array}$$

where *U*^*min*^ is the maximum theoretical utility achievable without any information processing and *U*^*max*^ denotes the maximal theoretical utility for a channel with an information processing rate of $D_{KL}^{exp}$. As the decision-maker's performance must lie either on or below the efficiency frontier, the efficiency obeys ϵ ≤ 1. Note that the efficiency can be equally measured in the space of actions *a* or in the space of consequences *x*, because 𝔼[*U*(*w, x*)] = 𝔼[*V*(*w, a*)].

### 2.2. Experimental Methods

#### Participants

Ten naïve subjects (5 female, 5 male) participated in the study and provided informed written consent prior to the participation. The study was approved by the ethics committee of Ulm University. They carried out the experiment over several days and were compensated for their time with a basic hourly wage of 8 € and 0-5 € bonus depending on performance given by their target hit ratio.

#### Setup

The experiment was conducted using a vBOT robotic manipulandum (Howard et al., 2009). Subjects moved the handle of the vBOT in the horizontal plane using their right hand. Position and velocity of the hand were recorded in each trial with a sampling frequency of 1 kHz during the performance of the movement task. A visual screen was projected into the plane of movement of the vBOT handle via a mirror. The virtual reality scenario provided a visual feedback about the hand position of the subject, preventing direct visual view of the hand.

#### Experimental Design

**Figure 2:**
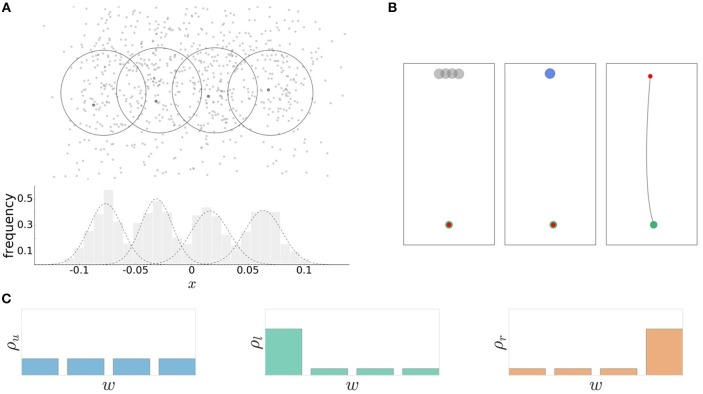
Experimental design. **(A)** The recorded reaching endpoints are distributed around the individual targets. The average endpoint lies close the target centers. The action consequences are approximated by normal distributions $p\left(x|w\right)=N\left(\mu_{w},\sigma_{w}^{2}\right)$. **(B)** Schematic sequence of the experimental task. Preparation phase: the cursor (red) is moved in starting position (green) and the four possible targets are shown. Stimulus presentation: the target is selected according to the world-state probability distribution. Movement execution: the movement is initiated within the permissable reaction time limit. The movement is executed within a fixed movement time interval of 300 ms resulting in a trajectory toward the target. **(C)** Three different probability distributions ρ_*u*_, ρ_*l*_, and ρ_*r*_ over the four targets correspond to different world state distributions.

In the experimental task, subjects had to perform reaching movements toward one of four concentrically located targets representing the world states $w\in W=\left\{w_{1},\ldots ,w_{4}\right\}$. Subjects' behavior was recorded as their reaching endpoints after a precisely defined time window for movement execution. The action consequence *x* was measured as a movement angle $x\in X=\left[\frac{-\pi }{2},\frac{\pi }{2}\right]$. As in previous studies (Trommershäuser et al., 2008), we distinguish between movement planning and movement execution and consider subjects' choices in the planning phase to be between different aim points *a*—also represented as an angular variable angle $a\in A=\left[\frac{-\pi }{2},\frac{\pi }{2}\right]$—that are related to the movement endpoints *x* through a distribution *p*(*x*|*a*) that considers the effects of noisy movement execution. In line with previous studies (Trommershäuser et al., 2003a,b), we found a roughly Gaussian distribution of movement endpoints (see Figure 2A).

***Trial design*. **The hand position was represented on the display by a red cursor with a diameter of 0.4 cm. In each trial, movement started from a home position with a diameter of 0.5 cm located 18 cm below the center of the screen. The desired endpoint of the movement was represented by a circular target which appeared at one of four fixed target positions. The target positions were set at a distance of 15 cm from the home position and had an angular distance of 2.5 ° to each other. Each target had a diameter of 1 cm and overlapped with neighboring target circles due to the small angular distance between targets. The overlap was roughly chosen at a distance where the difference between the mutual informations for different reaction times would be largest (see Figure S1). As the mutual information represents the identifiability of the target from subjects' responses, targets that are too close together cannot be distinguished, whereas targets that lie too far apart can always be distinguished independent of the reaction time limit. The intermediate distance that is most sensitive depends on each subject's execution noise level. For reasons of comparability, we chose the same angular distance for all subjects.

**Figure 3:**
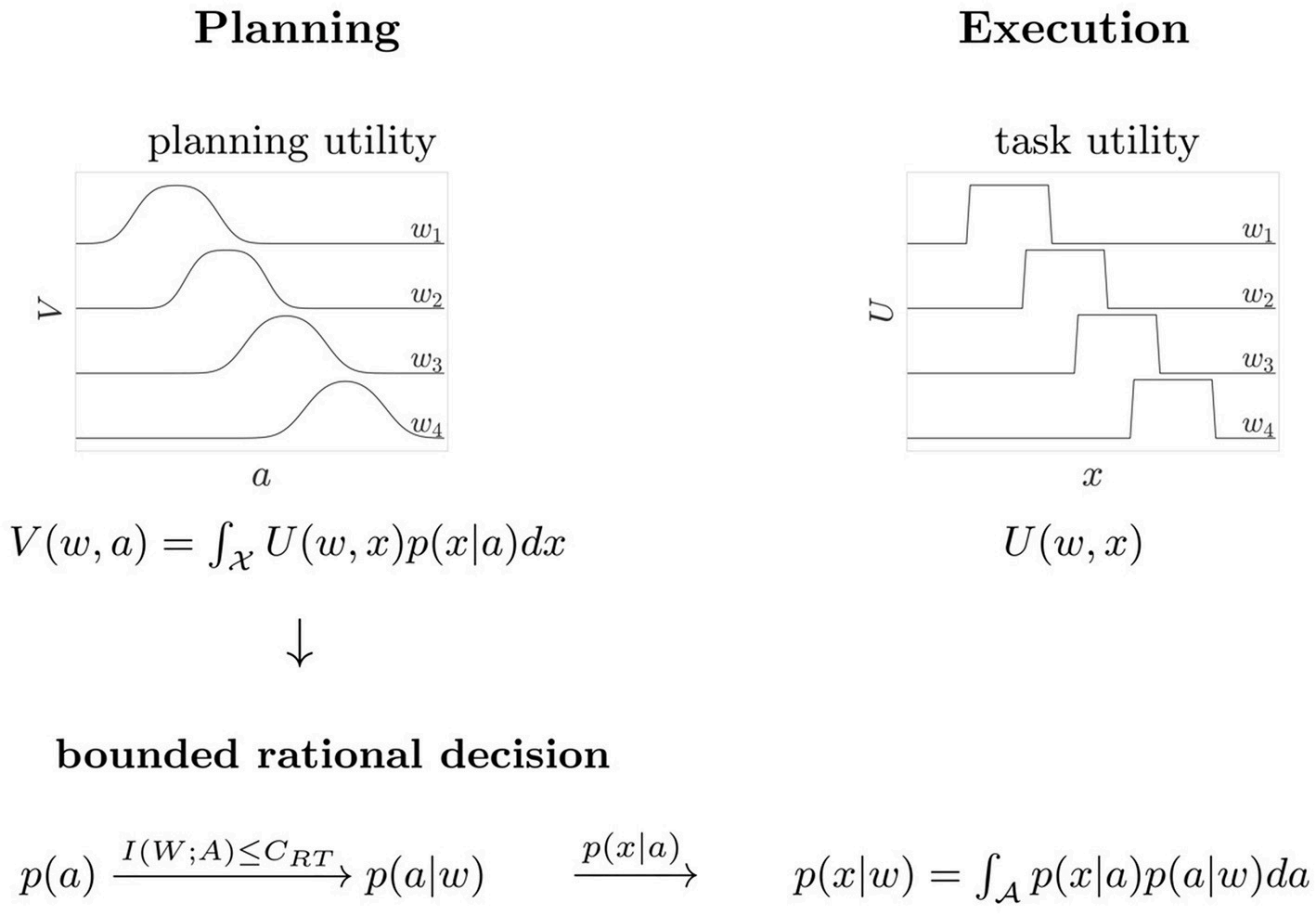
Scheme of the decision process. The task requires planning and execution of a movement. Based on the expected utility *V*(*w, a*) a bounded rational decision-maker makes a motor decision by sampling a movement aim point *a* from the distribution *p*(*a*|*w*) that maximizes *V*(*w, a*) for a given target *w*. During movement execution, the aim point *a* is corrupted by motor execution noise, resulting in the distribution *p*(*x*|*w*) of actual movement endpoints. The task utility *U*(*w, x*) indicates target hit or miss in the space of angular movement endpoints *x*.

Trials had a consistent flow of events (see Figure 2B). First, all four targets were displayed on the screen. After a 1 s hold period with the hand in the starting position, an acoustic signal resounded and a randomly selected target was highlighted and disappeared after movement onset. Subjects had to initiate the movement within a given reaction time limit after target presentation. The subsequent movement was restricted to a 300 ms time window. The recorded endpoint of the movement corresponded to the handle position at the point in time when the 300 ms expired. An auditory feedback provided information whether the target was hit, i.e., whether the recorded endpoint was inside the target circle. For our analysis in angular space, we counted any trial as a target hit in which the angle of the endpoint fell within the arc spanned by the target. This defines the 0/1-utility function *U*(*w, x*) ∈ {0, 1} as shown in Figure 3, where 1 corresponds to a target hit and 0 to a target miss. Trials where movement was initiated before target appearance or after the permissible reaction time limits were rejected and had to be repeated. Timeouts and premature reactions were displayed by a respective error message.

***Trial blocks*. **Subjects' behavior was tested in six different blocks where each block of 500 trials was characterized by a different condition. A condition was determined by the combination of the reaction time limit and the probability distribution ρ(*w*) over targets *w*. Two different reaction time limits *RT*_1_ and *RT*_2_ were defined. *RT*_1_ was intended to represent a hard time constraint which was set specifically for each subject. The second reaction time limit *RT*_2_ was the same for all subjects and defined a fixed limit of 300 ms. There were three different probability distributions over targets: uniform (ρ_*u*_), high probability for leftmost target (ρ_*l*_) and high probability for rightmost target (ρ_*r*_) (see Figure 2C). In total this results in six different conditions that were performed in random order. At the beginning of each block subjects were informed about the condition, i.e., they were told about the probability distribution over targets and the reaction time limits. Subjects' behavior in each condition was characterized by fitting a Gaussian distribution $p\left(x|w\right)=N\left(\mu_{w},\sigma_{w}^{2}\right)$ to subjects' posterior endpoint spread for each world-state. In order to measure subjects' movement execution noise in the absence of limitations in movement planning, the six conditions were followed by four blocks of 100 trials, each only to a single target with a reaction time limit of 1 s. In these trials we determined subject-specific movement precision by fitting another Gaussian $p\left(x|a\right)=N\left(a,{\tilde{\sigma }}_{w}^{2}\right)$, where ${\tilde{\sigma }}_{w}^{2}$ captures the subject-specific single target endpoint spread.

To allow for learning, the experiment was preceded by a training phase that consisted of four stages. In the first stage, the training phase started out with a block of 500 trials, where in each trial the target was selected randomly from a uniform probability distribution. To get subjects used to the task, the reaction time limit was set to 1 s. In the second stage, subjects underwent four blocks of 100 single target trials. As these four blocks were identical to the four blocks at the end of the experiment, one can get an idea about behavioral improvements of subjects. In the third stage of training, subjects' individual reaction time limit *RT*_1_ was determined, by fixing *RT*_1_ to a value well below 300 ms where approximately 20% of trials could not be completed in time. In the fourth and final stage of training, subjects underwent six blocks of 500 trials each, where each block was characterized by a different condition, identical to the test trials described above. Subjects' data across these blocks can be seen in Figure S2.

## 3. Results

The reaching task in our experiment consists of two processes, movement planning and movement execution (see Figure 3). In particular, we consider the planning phase as a decision-making process with limited resources, where the resources are given by the permissible reaction time and the amount of information implied by the prior probabilities. When faced with a randomly selected target stimulus *w*, subjects make a bounded rational decision by sampling a movement aim point *a* during motor planning so as to maximize the expected utility *V*(*w, a*) of hitting the target. The planning phase is followed by movement execution in which motor noise corrupts the planned aim point, which ultimately leads to an approximate Gaussian distribution of endpoints *x*. The movement endpoint is assigned the utility *U*(*w, x*) = 1 in case of a target hit and *U*(*w, x*) = 0 in case of a target miss. Note that the utilities for different world states *w* are overlapping in *x* because the targets are close together. From the bounded rational action selection *p*(*a*|*w*) and by taking into account execution noise, we can finally determine *p*(*x*|*w*), the distribution of movement endpoints under the bounded rational planning strategy. Unlike *p*(*a*|*w*), the distribution *p*(*x*|*w*) is directly observable. The two phases of movement planning and movement execution are associated each with their own type of noise, planning noise and execution noise. Execution noise is assumed to be roughly constant and irreducible for the time scale considered in our model, whereas planning noise can be modulated by allowing for different amount of planning resources. Experimentally, we distinguish between the two sources of noise by measuring endpoint variance in blocks of trials with a single fixed target, as this does not require any decisions or planning with respect to target selection. In line with previous studies (Trommershäuser et al., 2003a,b), we determined motor execution noise as the endpoint variance of movement angles *x* in such single target trials. The total endpoint variability in our experiment with multi-target trials, as illustrated for one subject and one condition in Figure 2A, is composed of both execution noise and planning noise. By manipulating permissible reaction time for movement planning, while leaving movement time constant (at 300 ms), we could therefore attribute any difference in total endpoint spread to planning noise.

### 3.1. Resource Manipulation I: Varying Reaction Time

**Figure 4:**
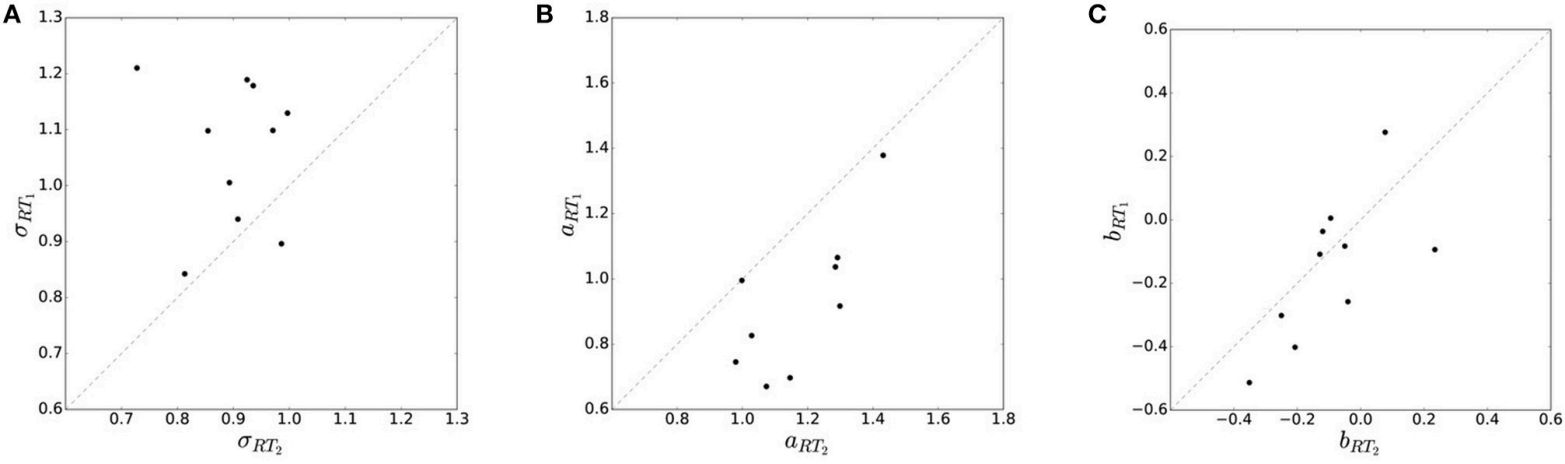
Effect of resource limitation on movement variability. **(A)** Movement variability in the two reaction time conditions is measured by subjects' standard deviation σ. Standard deviation is generally higher (i.e., precision is lower) in the faster reaction time condition *RT*_1_. **(B)** Movement accuracy *a*_*RT*_ in the two reaction time conditions is measured by deviation from the correct target location. Accuracy is generally higher in the slower reaction time condition *RT*_2_. **(C)** Subjects' movement bias shows no significant trend across different *RT* conditions.

We compared two reaction time conditions for each subject: a fast condition and a slow condition, *RT*_1_ and *RT*_2_ respectively. Averaged over all subjects we measured a mean reaction time τ_*R*_*T*__1__ = 154 ± 10 ms and τ_*R*_*T*__2__ = 202 ± 5 ms. By manipulating the permissible reaction time, we effectively manipulated subjects' decision-making resources. According to bounded rational decision theory one would expect higher decision noise with less resources, and therefore an increased total endpoint variance in case of *RT*_1_ compared to *RT*_2_. In line with our prediction we found a general tendency of increased movement variability when decreasing subjects' reaction time limit. Figure 4 compares subjects' movement variance, accuracy and bias in the different reaction time conditions *RT*_1_ and *RT*_2_ for uniform target distribution ρ_*u*_. In particular, we find that standard deviation σ = 〈_σ_*w*_〉ρ(*w*)_ of subjects' endpoints on average increased when decreasing subjects' reaction time limit (*p* = 0.0047, repeated measures ANOVA) (see Figure 4A). In other words, movement precision measured by the inverse of the standard deviation decreased with decreasing resources. Subjects' average movement accuracy *a*_*RT*_: = ${\langle {\left[\frac{1}{N}\underset{i}{\sum }{\left(x_{i}-x_{w}^{*}\right)}^{2}\right]}^{-\frac{1}{2}}\rangle }_{\rho \left(w\right)}$ measured as the mean squared deviation of movement endpoints from the desired target location $x_{w}^{*}$ is significantly higher in the slower reaction time condition (*p* = 0.0042, repeated measures ANOVA) (see Figure 4B). Movement accuracy is related to movement variability by $a_{RT}={\langle {\left(\sigma_{w}^{2}+b_{w}^{2}\right)}^{-1/2}\rangle }_{\rho \left(w\right)}$, where the bias *b*_*w*_ represents the difference between mean movement endpoint and desired target location $x_{w}^{*}$ . While we find significant effects for movement variability and accuracy, we find no systematic effects in the average movement bias *b* = 〈_*b*_*w*_〉ρ(*w*)_ (see Figure 4C).

To ensure that differences in movement planning were indeed the major explanans for the observed differences in endpoint variability, we checked that movement execution was similar for the two reaction time conditions. We therefore looked at trajectory paths, velocity profiles and trajectory variance averaged over all of subjects' movements. While we found no systematic differences between movement paths in the fast and slow reaction time conditions (see Figure S3), we found that movement variance was considerably higher in trials with faster reaction time, which is reflected in the endpoint variability shown in Figure 4. However, we also found that the faster reaction time condition was characterized by slightly higher peak velocity in the movement, on average 4% faster than the slow reaction time condition (see Figure S4). Yet, this elevated movement velocity cannot explain the differences in variability. This can be seen when we select trajectories in the slow reaction time condition based on their peak velocity and only allow trajectories that have a peak velocity that lies in a band of width *b* with respect to the mean velocity of the fast reaction time condition. The width *b* was determined as the absolute difference between the peak velocities in the fast and slow reaction time conditions. With this selection of trajectories, there is no significant difference between peak velocities in the two reaction time conditions (*p* = 0.847, repeated measures ANOVA). Importantly, however, while the difference in peak velocities vanishes, the difference in variability remains (*p* = 9.092·10^−5^, repeated measures ANOVA) as can be seen in Figure S5. We can therefore conclude that differences in endpoint variability are indeed mostly due to differences in motor planning and not motor execution.

**Figure 5:**
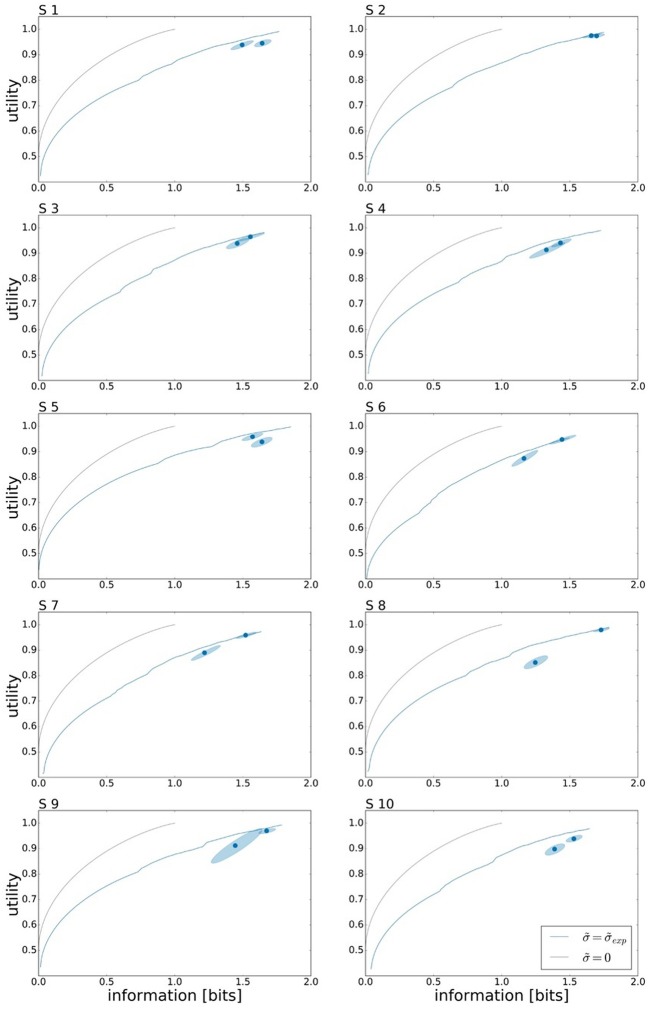
Bounded rational analysis of movement performance for different reaction times and uniform target distribution ρ_*u*_. Movement performance is given by subjects' average target hitting probability (expected utility) and sensorimotor information in bits measured by the mutual information *I*(*W*; *X*) between movement endpoints and world state. The two data points correspond to the movement performance measures for the two different reaction times *RT*_1_ and *RT*_2_. The 95 % confidence region is determined by bootstrapping. Subjects' experimental performances are each compared against two bounded optimal efficiency frontiers, arising as the theoretical predictions with and without execution noise. For planning in an ideal system without movement execution noise ($\tilde{\sigma }=0$) we obtain a steeper efficiency frontier than for bounded optimal planning by taking into account subjects' individual execution noise levels (${\tilde{\sigma }}_{exp}$) measured in single target trials.

To assess the effect of limited reaction time on subjects' performance, we investigate the change in utility and information. Subjects' overall performance is measured by their expected utility 𝔼[*U*(*w, x*)] under the world state distribution ρ(*w*) and the Gaussian strategy *p*(*x*|*w*) fitted to the subject's responses, and their planning effort by the effective information measure *I*(*W*; *X*) that determines how specific subjects' actions are for each target—more specific actions, require more planning. Subjects' individual performances for both reaction time limits under ρ_*u*_ can be seen in Figure 5 represented by the two data points. The precise utility and information values of all the data points can be found in the Tables S1, S2 respectively. On average over all subjects we measured a mean information of *I*_*R*_*T*__1__ = 1.42±0.05 bits and *I*_*R*_*T*__2__ = 1.56±0.03 bits. Forming the difference quotient between information and reaction time, we get a mean information rate of approximately 3 bits/s.

In Figure 5 the performance measurements for both reaction time conditions are compared against the optimal performance of a bounded rational decision maker. Here, we consider two theoretical predictions of planning the optimal actions with and without taking into account execution noise. In the limit of infinite planning resources, a bounded rational decision-maker in the absence of execution noise ($\tilde{\sigma }=0$) would be able to pick any endpoint with arbitrary precision and therefore reaches the maximum utility of 𝔼[*U*(*w, x*)] = 1. This maximum is reached at the expenditure of 1 bit, as the optimal prior is set at the two overlapping areas between targets *w*_1_ and *w*_2_ and between targets *w*_3_ and *w*_4_, such that the posterior simply selects one of the two possibilities. When considering execution noise in the planning phase, the optimal performance of a bounded rational decision-maker depends on the individual execution noise level ${\tilde{\sigma }}_{exp}$ that determines the expected utility *V*(*w, a*) that is used for planning (see Figure 3). It should be emphasized that the precise shape of the optimal performance curve for each subject is affected by the experimentally measured level of motor execution noise (see Figure S6). Such a bounded rational decision-maker requires more information resources to reach the same level of utility than a bounded rational decision-maker without execution noise. Also such a decision-maker, may not achieve a maximum utility of 𝔼[*U*(*w, x*)] = 1 even for planning with unlimited resources. At the other extreme, without any information processing [i.e., *I*(*W, A*) = 0], the posterior strategy cannot deviate from the prior. When comparing the two theoretical predictions, it can be seen that subjects' performance is much closer to the theoretical prediction considering execution noise, i.e., performance always lies slighly below the bounded optimum under execution noise, and well off the bounded optimum without execution noise.

**Figure 6:**
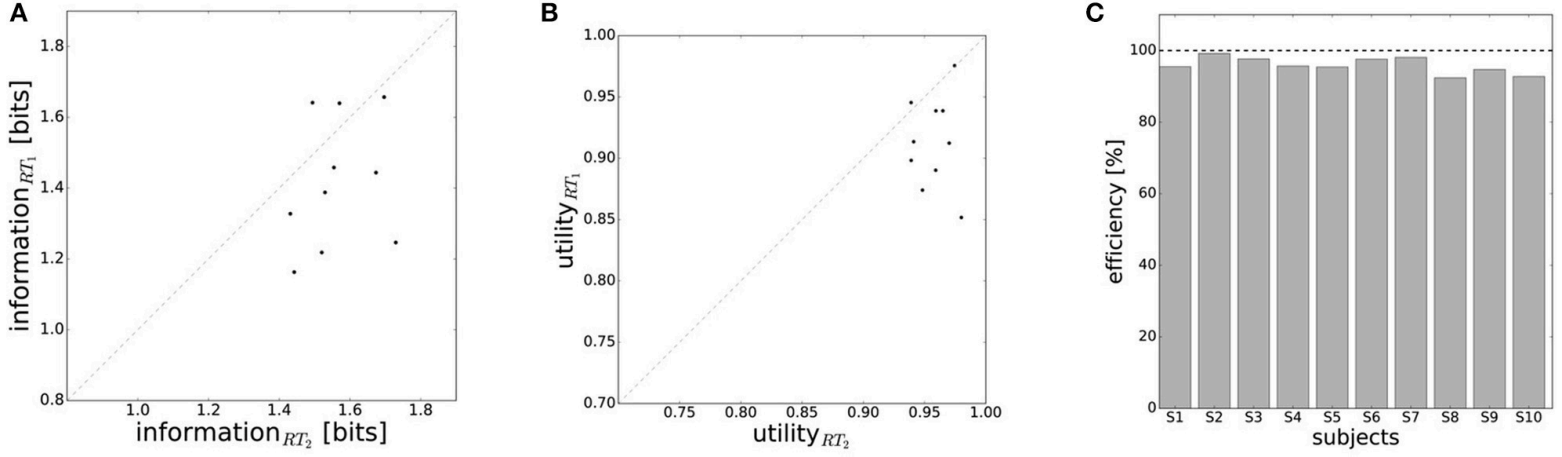
Performance with different reaction time resources (*RT*_1_<*RT*_2_). **(A)** A limitation in reaction time resource results in a decrease in information resources. **(B)** With less resources available, decision-makers achieve less expected utility. **(C)** Subjects' average efficiency is generally high (above 92%).

In line with the prediction of Figure 1A, we find that for most subjects a lower reaction time limit produces behavior with lower expected utility and less information processing. The difference in information and utility caused by different reaction time limits is shown in Figure 6. As expected, the amount of information resources *I*(*W*; *X*) decreases when given less reaction time in condition *RT*_1_ (*p* = 0.042, repeated measures ANOVA) , which can be seen in Figure 6A. Similarly, the utilities 𝔼[*U*(*w, x*)] are significantly lower when given less reaction time in condition *RT*_1_ (*p* = 0.0028, repeated measures ANOVA), which can be seen in Figure 6B. Generally, subjects were more efficient in the slower reaction time condition *RT*_2_, even though average efficiencies as defined in Equation 8 are well above 90% throughout for all subjects (see Figure 6C).

### 3.2. Resource Manipulation II: Varying Prior by Changing World State Distribution

**Figure 7:**
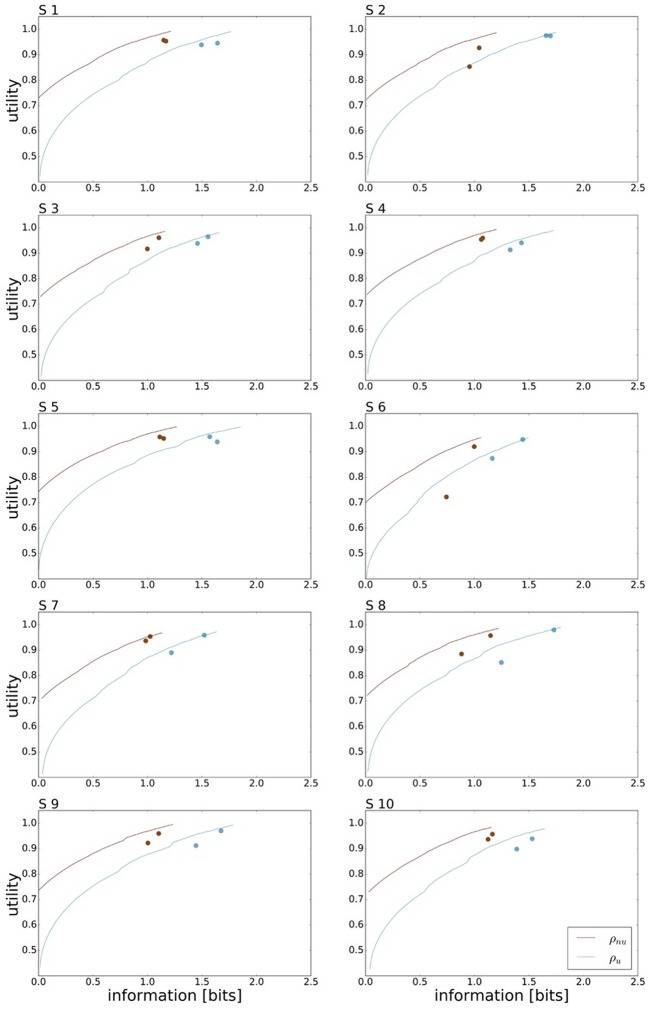
Bounded rational analysis of movement performance for varying world state distributions. Subject's experimental performances are each compared against the bounded optimal efficiency frontier for given world state distributions. For the non-uniform world state distribution ρ_*nu*_ the information processing cost is reduced compared to the uniform distribution ρ_*u*_.

A bounded rational decision-maker should consider the given world state distribution ρ(*w*), as this affects the optimal action prior p^*^(*a*). Consequently, the amount of mutual information needed for a bounded rational decision-maker to gain maximum expected utility depends on the given world state distribution. In our case, for the uniform world state distribution ρ_*u*_ the processing cost to achieve maximum expected utility is approx. 1.7 bit for an average level of motor execution noise. In comparison, the non-uniform distribution ρ_*nu*_ that represents the mirror symmetric distributions ρ_*l*_ and ρ_*r*_ from Figure 2C allows for planning with less ressources as predicted in Figure 1B. In our case, we expect approx. 1.2 bits for maximum performance. Subjects' performance under the non-uniform target distribution ρ_*nu*_ is depicted in Figure 7, where we have averaged the performances recorded for ρ_*r*_ and ρ_*l*_. Subjects' performance in the two reaction time conditions is compared against the bounded optimal efficiency frontier for ρ_*nu*_ considering the individual level of movement execution noise. On average over all subjects we measured mean reaction times of τ_*R*_*T*__1__ = 155 ± 9 ms and τ_*R*_*T*__2__ = 189 ± 6 ms and mean information values of *I*_*R*_*T*__1__ = 1.01±0.03 bits and *I*_*R*_*T*__2__ = 1.09±0.02 bits. Taking the difference quotient of information and reaction time we estimate an information rate for the population of approximately 2.1 bits/s, which is similar to the uniform condition.

**Figure 8:**
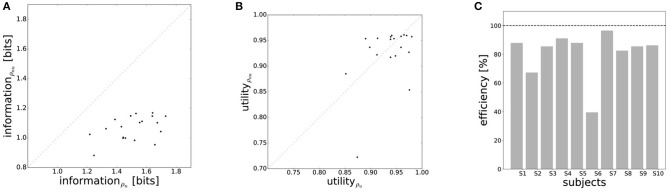
Performance under non-uniform world state distributions. **(A)** A more concentrated world state distribution effectively results in a decrease in information. **(B)** Decision-makers' expected utility is not affected by a change in world state distribution. **(C)** Subjects' average efficiency for ρ_*nu*_ is generally lower than for ρ_*u*_.

The efficiency frontier for the non-uniform distribution ρ_*nu*_ is shifted to the left compared to the efficiency frontier under the uniform world state distribution ρ_*u*_. As predicted in Figure 1B, we observe that the data points are shifted to the left compared to the data points under the uniform world state distribution. The changes in information and utility for varying world state distributions are shown as a scatter plot in Figure 8. As expected, the amount of mutual information *I*(*W*; *X*) decreases when given less uncertainty about the world state (*p* = 1.48·10^−8^, repeated measures ANOVA)(see Figure 8A). In contrast, the utilities 𝔼[*U*(*w, x*)] do not change significantly when changing the world state distribution (*p* = 0.579, repeated measures ANOVA)(see Figure 8B). Generally, subjects were less efficient in the non-uniform condition (see Figure 8C), which may be a consequence of incomplete adaptation to the non-uniform world state distribution.

**Figure 9:**
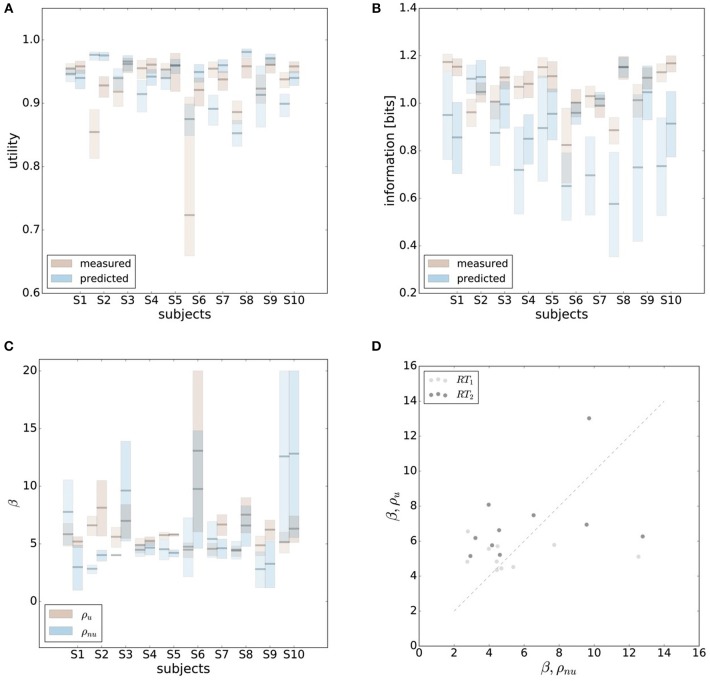
Predicting performance across world state distributions. **(A)** Predicting confidence bounds for utility in non-uniform condition from uniform condition by assuming that utility levels are maintained. **(B)** Predicting confidence bounds for information in non-uniform condition from uniform condition by assuming that utility levels are maintained. **(C)** Confidence bounds for beta estimates in uniform and non-uniform condition. **(D)** Means of β intervals shown in **(C)**.

In order to make predictions about subjects' performance for non-uniform world state distributions from their behavior under a uniform condition, it is necessary to make additional assumptions about the constraints that determine the decision-making process. For the prediction of the bounded rational decision-making model in Figure 1B, for example, we assumed that subjects achieve the same level of utility under both world state distributions. The results presented above are in line with this assumption, as we found that on average there was indeed no systematic deviation in utility between the uniform and non-uniform condition (see Figure 8B). On the basis of this assumption we predict for each individual subject the level of utility and the effective information in the non-uniform condition from the 95%-confidence intervals of utility and information in the uniform condition (see Figure 9). For 7 out of 10 subjects, we find an overlap in the confidence intervals of the measured utilities for ρ_*u*_ and ρ_*nu*_ in both reaction time conditions (see Figure 9A). Only Subject S2 differs in utility with non-overlapping confidence bands in both reaction time conditions when changing the world state distribution. Similarly, we compared actual and predicted confidence bounds for subjects' information resources under a constant utility constraint. The predicted confidence bound for information was determined by projecting the two bootstrap confidence bounds of the utilities in the uniform condition to the non-uniform efficiency frontier (see Figure 9B). Without considering inefficiencies, the predictions underestimate the information systematically. When allowing for inefficiencies at the average measured ineffiency level of 86%, 9 out of 10 subjects have overlapping confidence bounds for measured and predicted information resources.

**Figure 10:**
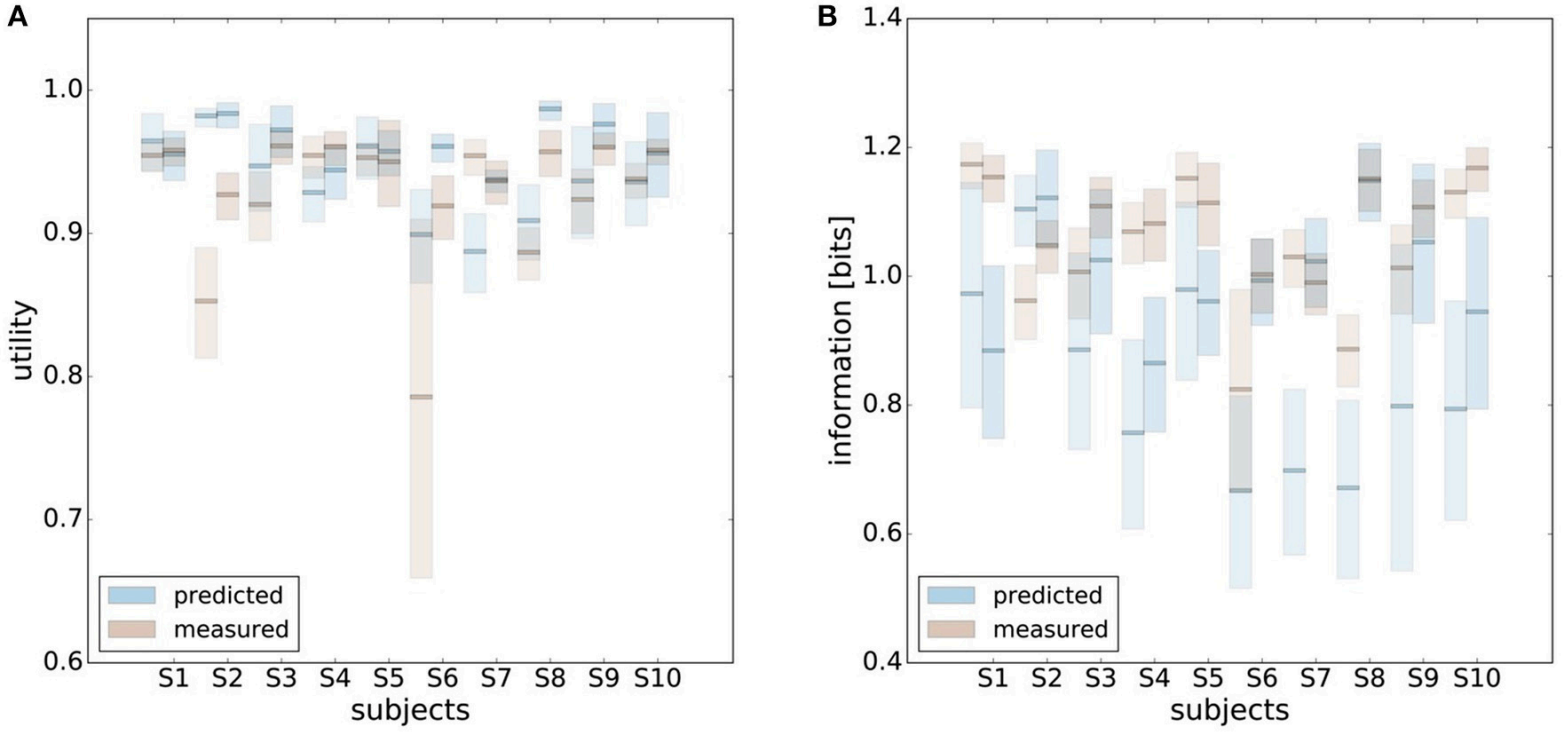
Comparison of predicted and experimental utility and information when predictions are generated from the uniform condition under the hypothesis that the rationality parameter β is constant. **(A)** shows confidence intervals for utility values belonging to a range of possible β-values assigned to a data point, as described in the main text. For 6 out of 10 subjects the confidence intervals overlap in both reaction time conditions, i.e., prediction matches experimental measurement. **(B)** shows confidence intervals for information for the same range of β-values. Predicted information is only considered for bounded optimal actors, which is why there is not much overlap in confidence intervals. However, when allowing for inefficiencies as described in the main text, hypothesis (2) cannot be ruled out.

**Figure 11:**
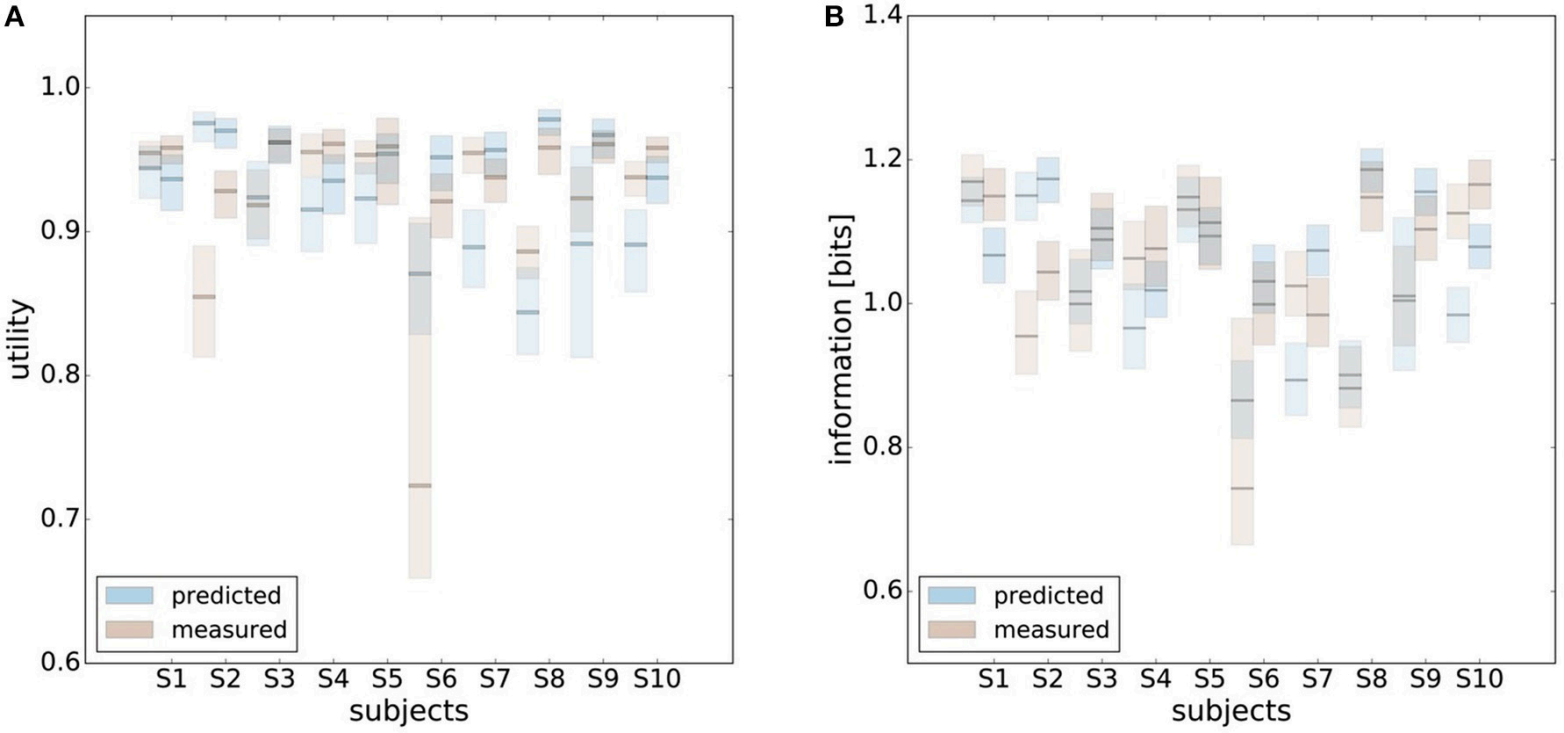
Comparison of predicted and experimental utility and information when predictions are generated from the uniform condition under the hypothesis that there is no adaptation and the posterior *p*(*x*|*w*) is constant. **(A)** shows confidence intervals for utility values belonging to different posteriors fitted to a set of bootstrapped data. For 7 out of 10 subjects the confidence intervals overlap in both reaction time conditions, i.e., prediction matches experimental measurement. **(B)** shows confidence intervals for information of the same fits. In this prediction inefficiencies are implicitly considered, which is why there is a higher overlap.

**Figure 12:**
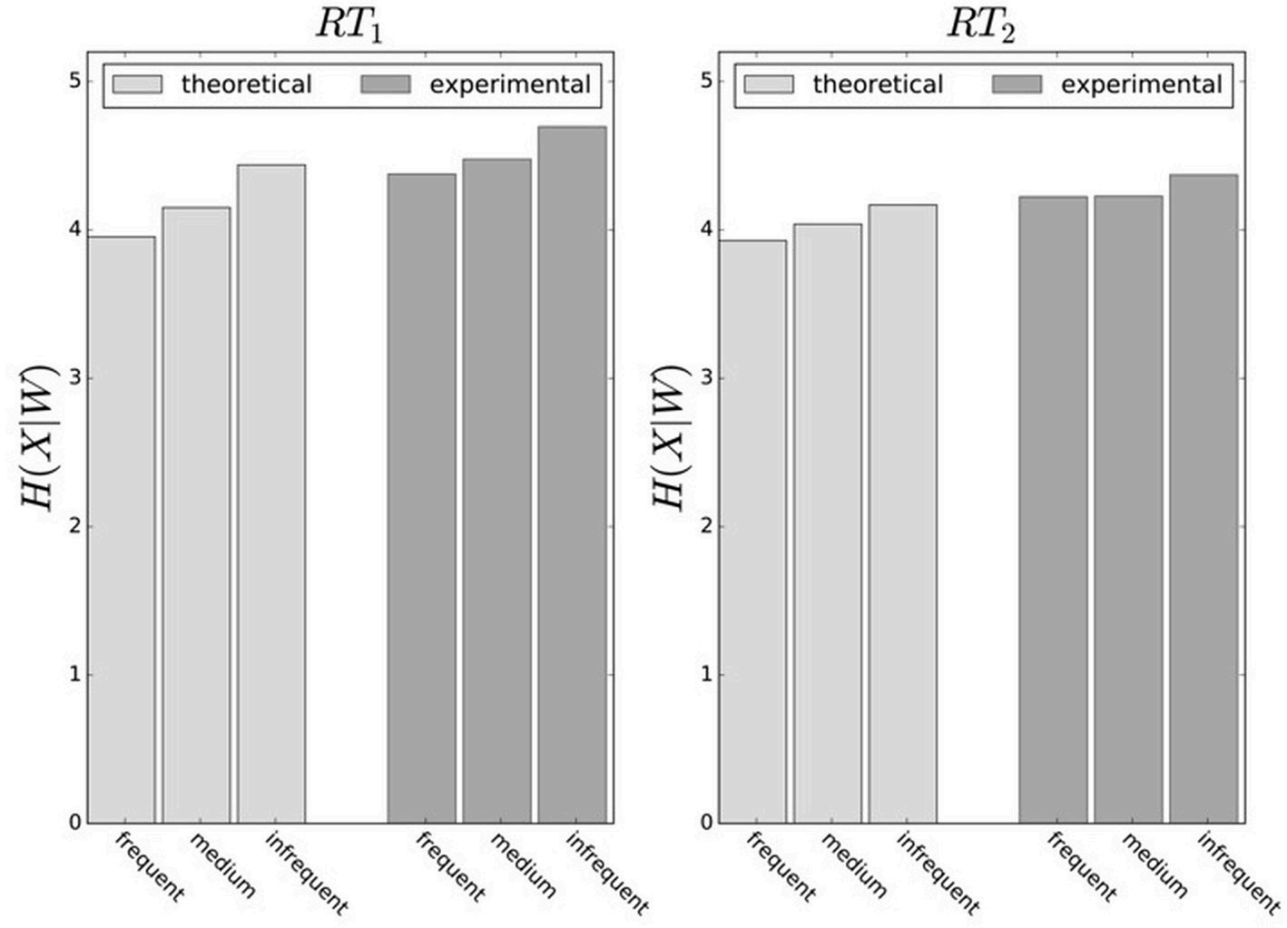
Conditional entropy *H*(*X*|*W*). Average entropy of endpoint distribution conditioned on the world state. World states are summarized as frequent, medium and infrequent. High-frequent world states have lower entropy, because endpoint spread is smaller. Experimental entropies are averaged over all subjects and obtained from measured posteriors *p*(*x*|*w*), theoretical model predictions are for bounded optimal posteriors p^*^(*x*|*w*) under a constant utility hypothesis.

In principle, there are alternative assumptions one could make to predict behavior in the non-uniform condition from uniform performance. We consider three possible alternatives: (1) one could try to achieve a higher utility by maintaining the same level of information, (2) one could reoptimize prior and posterior by maintaining the same rationality parameter β, and (3) one could simply maintain exactly the same behavior as before without adapting at all. The first hypothesis can be ruled out immediately when looking at Figure 7. Since the maximum information under the non-uniform condition is lower than the actual information in the uniform condition, one would expect all subjects to be able to perform at the maximum information level and very close to 100% utility. This is obviously not the case and alternative hypothesis (1) can be rejected. As we will elaborate in the discussion, the rejection of hypothesis (1) is not surprising, as the mapping between concrete resources and information changes with the world state distribution. The second alternative hypothesis with constant β would predict in our case that utilities should remain roughly the same and that information resources should decrease (see Figure 10), which is in quantitative agreement with our experimental data. To distinguish better between this hypothesis and the other alternatives, we therefore attempted to extract estimates of the rationality parameter β for each subject. Strictly speaking this is only possible for data points that lie exactly on the efficiency frontier. However, as subjects were in general very close to the efficiency frontier we determined the rationality parameters of the bounded optimal actors nearby that were closest. To this end, for each data point we considered all the β-values as neighbors that would lie between the bounded optimal actor with the same utility level as the data point, but with lower (optimal) information, and the bounded optimal actor with the same information resource level, but with higher (optimal) utility. The extracted interval of β-parameters can be seen in Figure 9C. While it seems that there is a tendency for β to be higher in the uniform condition, this trend is not significant (*p* = 0.477, repeated measures ANOVA). We can therefore not rule out hypothesis (2). The third alternative hypothesis without adaptation would also predict that utilities should remain roughly the same and that information resources should decrease (see Figure 11). To obtain this prediction we simply assumed that the experimental posterior *p*(*x*|*w*) fitted in the uniform condition would stay exactly the same in the non-uniform condition. To better distinguish this hypothesis from our constant utility hypothesis, we studied the conditional entropy of the posteriors more closely. As shown in Figure 1C we expect that the conditional entropy of action distributions *H*(*A*|*W* = *w*_*i*_) for more probable world states *w*_*i*_ is lower than entropy of action distributions for less probable world states *w*_*i*_. Figure 12 compares the theoretical entropy predictions with action entropies from the experiment. The frequency terms *frequent, medium, infrequent* arise from the three different world-state distributions ρ_*u*_, ρ_*l*_ and ρ_*r*_ and relate to the outer most target locations (*w*_1_, *w*_4_). The theoretical entropies are computed from the bounded optimal posteriors that have the same expected utility as the experimental posteriors. For frequent targets we average the theoretical entropies for target *w*_1_ in the case of ρ_*l*_ and target *w*_4_ in the case of ρ_*r*_ and compare against the experimentally determined action entropy for the same targets. For infrequent targets we average the theoretical entropies for target *w*_4_ in the case of ρ_*l*_ and target *w*_1_ in the case of ρ_*r*_ and compare against the experimentally determined action entropy for the same targets. The *medium* frequency comprises both target *w*_1_ and target *w*_4_ under the uniform target distribution ρ_*u*_. Dependent on the world state frequency the optimal entropy of the action distribution is modulated, such that frequent world states should be associated with lower entropy and infrequent world states with higher entropy. This way the average entropy is lowered. The experimental results confirm the trend that the action entropy decreases with increasing world state frequency (*RT*1:*p* = 0.037, *RT*2:*p* = 0.024, repeated measures ANOVA). While this result fits well with the constant utility and constant β hypotheses, the third alternative hypothesis with constant posterior has to be rejected, as this would predict that the conditional entropy should not change depending on the world state distribution.

## 4. Discussion

In this study we investigated how the abstract theory of information-theoretic bounded rationality can be applied to a sensorimotor reaching experiment by varying informational resources during motor planning. In particular, we varied the permissible reaction time for planning and the probability distribution over the different targets to manipulate subjects' action prior. We found that both information constraints had a significant impact on task performance as measured by endpoint variability and expected utility (i.e., the expected probability of a target hit under known motor execution noise). The two experimental manipulations of reaction time and prior can be mapped into abstract informational resources quantified by relative Shannon information within bounded rationality theory. Our results show that a decrease in permissible reaction time is accompanied by a decrease in both utility and information-processing resources, and that changing to a low-entropy world state distribution also decreases information-processing costs, as the motor system selectively adapts endpoint variability to the probability of the targets. Both the reduction in information-processing due to reaction time limits and the modulation of endpoint variability depending on target frequency can be understood within the normative framework of bounded rationality. Usually, optimal decision-making models under motor execution noise either assume perfect planning or add planning noise in an *ad hoc* fashion to fit the data. In contrast, bounded rationality models allow to compare performance against the best possible performance with a given information resource in a similar way that information theory allows to compare the performance of codes to the theoretical optimum. We found that the experimental behaviors were generally close to bounded optimal. The reported inefficiencies are small and due to biases in behavior that are not considered in the priors and utilities of the model. Investigating the source of such inefficiencies may constitute an interesting avenue of future research that is opened up by such a framework.

Bounded rationality with information-theoretic concepts has been investigated in the context of economic decision-making (Sims, 2003), and has also been applied to high-level cognitive problem solving and perceptual decision-making (Ortega and Stocker, 2016; Sims, 2016). Here we chose to apply information-theoretic bounded rationality concepts to a very basic sensorimotor reaching task, on the one hand to emphasize the generality of the framework and its applicability to any kind of behavior, on the other hand because one might rightly expect such highly trained behavior to be efficient and close to bounded optimal. A consequence of choosing such a basic task is that information-processing is extremely fast and stereotyped, which makes it difficult to measure behavior at different resource levels. We found, for example, in our task that already at a “slow” reaction time limit of 300 ms, subjects did not benefit from looser limits, and in fact their average reaction time in these trials was far below the limit at approximately 200 ms. In contrast, the subject-specific “fast” limit was set between 190 ms and 220 ms with average reaction times at 150 ms, which is fairly close to the slow condition. Introducing a third level between slow and fast would have little extra value, as the theoretically maximal differences we can expect in mutual information are already rather small (see Figure S1). Lowering the reaction time limit below the fast limit is also fraught with problems, because on average over all blocks subjects already had to repeat 18% of trials when they initiated movements too late. Increasing the failure rate further would have been difficult both in terms of keeping subjects motivated and in terms of the required number of trials (currently 7, 300 trials per subject spread over approximately 10 h). Ultimately, this lower bound is of course not too surprising, as it is impossible to execute a movement in the total absence of motor planning, even if it is totally random (i.e., it generates zero relative information), because there is a minimal time for information-processing that constitutes an offset.

### Information-Theoretic Models of Sensorimotor Processing

Two of the earliest applications of information theory to human behavior were Hick's law (Hick, 1952) and Fitts' law (Fitts, 1954). Hick measured reaction time in simple choice reaction time experiments and found a linear relationship between choice reaction time *RT* and the logarithm of the number *n* of options, such that *RT* = *a* + *b*log(*n* + 1). This law can be considered as a special case of *RT* = *a* + *bI*(*n*), where *I*(*n*) is the Shannon information under arbitrary stimulus distributions (Hyman, 1953). Hence, according to Hick-Hyman's law there is a linear map between information and time resources for decision-making during motor planning, typically in tasks with constant accuracy requirements where subjects choose their preferred reaction time. In contrast to Hick's reaction time study, Fitts investigated a speed-accuracy trade-off in motor execution by measuring movement time *MT* in a reciprocal tapping task as a function of target width *W* and target distance *D*, and found that $MT=a+b\log\frac{2D}{W}$. In analogy to Shannon's channel capacity *C* for a Gaussian channel with bandwidth *B* and signal-to-noise ratio *S*/*N* given by $C=B\log\left(\frac{N+S}{N}\right)$, Fitts equated his index of difficulty $ID=\log\frac{2D}{W}$ with the logarithm of the signal-to-noise ratio, which represents the amount of information in bits that needs to be processed. In this interpretation, there is again a linear relationship between time and information resources in a task with constant accuracy requirement where subjects choose their preferred movement time.

Our experiment could be interpreted as a Hick's experiment in continuous space. For experimental design reasons we decided not to have continuous targets, so we could average responses for each target without making a global translational symmetry assumption. Instead, we chose four targets as an approximation to the continuous case. For a continuous uniform target distribution $\rho \left(w\right)=\frac{1}{w_{\max}-w_{\min}}$ and Gaussian endpoint distribution $p\left(x|w\right)=N\left(w,\sigma^{2}\right)$ the mutual information would be

$$\begin{array}{r} \begin{array}{l} I\text{ ( }W\;;\;X\text{ ) = }H\;(\;W\;)\;-\;H\;(\;W\;|\;X\;)\\ \;=\log\left(w_{\max}-w_{\min}\right)-\frac{1}{2}\log(2\pi e\sigma )+f\left(w_{\min},w_{\max},\sigma \right), \end{array} \end{array}$$

where the first term is the differential entropy of the target distribution, the second term is the differential entropy introduced by Gaussian noise and the third term is a correction term due to truncation. For our approximation the first and second term would stay the same, the third term would vary depending on target distance and number of targets. In Figure S7, one can see that for the range of information values considered in our experiment, there is an equivalent continuous interpretation. In contrast, Hick considered discrete target and action spaces, where mutual information is obtained from the probability of target hit or miss. If we had classified actions into discrete categories like Hick and ignored actions that missed all targets, we would have obtained the following discrete information values:

$$\begin{array}{r} \begin{array}{l} \text{ uniform condition: }I_{RT_{1}}=1.91\pm 0.02\text{ bits and }\\ \;I_{RT_{2}}=1.95\pm 0.01\text{bits}\\ \text{ non-uniform condition: }I_{RT_{1}}=1.29\pm 0.03\text{ bits and }\\ \;I_{RT_{2}}=1.26\pm 0.02\text{ bits. } \end{array} \end{array}$$

Information values for both world state distributions are very close to their respective maxima of $I_{u}^{\max}=2$ bits and $I_{nu}^{\max}=1.36$ bits, which suggests that subjects performed nearly perfectly in terms of aiming for the correct target in angular space. In a conventional discrete reaction time analysis as pioneered by Hick, one would conclude that the task was too simple, because subjects always achieved plateau performance, which is reflected in the fact that the information values across the two reaction time conditions are virtually the same. However, in terms of continuous endpoint spread we observed a clear difference in information between reaction time conditions, which highlights that such a continuous analysis provides further insight compared to the discrete analysis championed by Hick.

Another important difference from Hick's task to our experiment is that we had a hard controlled reaction time limit, whereas the orignal experiment was a free reaction time task with a given accuracy level. In follow-up studies, for example by Pachella and Fisher (1972), the relationship between reaction time and information was also studied in speed constraint conditions and found to be of roughly linear shape, although the coefficients of this relationship depend on the number of alternatives and the linear shape levels off for high reaction times. Our results are consistent with these previous findings in that for the same reaction time subjects generate different levels of information depending on the world state distribution—where world state distributions with different entropy effectively correspond to different number of alternatives. This is exactly what one would expect from a bounded rationality perspective, namely that for each world state distribution there is a monotonic relationship between information and resource (e.g., time) and that for different world state distributions this relationship will change, because the range of possible information values changes. In general, the monotonic shape would be expected to be marginally decreasing, as more and more samples or resources are necessary to increase mutual information which will ultimately plateau at its maximal value for a given world state distribution. A roughly linear relationship would hold in the initial slope before information plateaus and only in special cases across the entire range (e.g., in case of logarithmic search). In contrast to this time-information relationship for each particular world state distribution, Hick's law relates the maximum information in the plateau (where there is no reaction time constraint) across different world state distributions with different entropies and number of states.

As we measured endpoint accuracy in reaching movements, Fitts' law is of course in principle applicable to our task. However, Fitts' law doesn't say anything about reaction time which we manipulated, but only about movement time which we did not manipulate. As we kept movement time, target distance and target width constant throughout the experiment, Fitts' law cannot explain our changes in endpoint accuracy due to reaction time manipulation. As suggested previously (Albertas et al., 2012), Fitts' law would need to be extended to encompass reaction time as a variable. While Fitts himself considered reaction time as a parameter in a follow-up study (Fitts and Peterson, 1964), the results were interpreted as showing only weak effects of reaction time (compared to movement time), suggesting a strictly serial information-processing pipeline, and therefore reaction time never got integrated into Fitts' law. In the future one could design experiments where both reaction time and movement time are varied to develop a general information processing law where both Hick's law and Fitts' law would be special cases.

How does bounded rationality contribute to this line of study? While our experimental design can be interpreted as a Hick's experiment in continuous space, both Hick's law and Fitts' law are fundamentally concerned with the relationship between information and time. In contrast, bounded rationality studies the relationship between utility and information, where information is conceived as an abstract resource measure, ultimately counting how many distinctions one can make. In general, one would expect a monotonic relationship between information and any concrete resource measure like time, because the more resources are available the more distinctions one can make (for example, the longer the computation of the number pi, the more digits are known). A linear relationship between information and resource would only be expected in special cases, for example in case of logarithmic search. In this sense both Hick's law and Fitts' law are consistent with bounded rationality theory, even though these findings only concern the resource part and ignore utility. In contrast, within a bounded rationality framework one could study effects of different utilities on the choice task, for example in the form of monetary payoff or by changing features of the stimulus. Norwich et al. (1989) for example has investigated the effect of stimulus features on reaction time, especially stimulus intensity of a single stimulus, by assuming that a subject requires a certain amount of information Δ*H* before they can react to the stimulus, where Δ*H* increases by the number of times a receptor is allowed to sample the environment. In the future it would also be interesting to consider such manipulations in a bounded rational framework. This illustrates how bounded rationality allows asking questions beyond the realm of Fitts' and Hick's law.

### Computational Models of Imperfect Decision-Making

As far as the authors are aware, this study is the first to show the effect of limited reaction time on continuous endpoint variability in a human reaching task. There is a large body of studies that have investigated speed-accuracy trade-offs (Wickelgren, 1977; Chittka et al., 2009) using reaction-time experiments to shed light on the dynamics of neural information processing, in particular the effect of noisy motor planning on behavioral accuracy in discrete choice tasks. In an early study by Schouten and Bekker (1967), for example, it was found that reaction times are shorter when subjects are asked to speed up their response, which, however, results in more errors. This is similar to Hick's choice task, except that Hick had a free reaction time choice task and simply encouraged subjects to allow for more errors. Both tasks do not consider endpoint accuracy but discrete choice accuracy. Endpoint accuracy is considered in Fitts' studies as part of the movement difficulty ID. While Fitts did not investigate reaction time in his original study, in a follow-up study Fitts and Peterson (1964) also studied effects of movement difficulty on reaction time and movement time in discrete target reaches. When manipulating the probability distribution over targets, Fitts and Peterson (1964) found that reaction time and movement time for frequent targets was reduced. He also found that accuracy somewhat increased depending on target frequency, similar to our finding in Figure 12. However, Fitts' task differed from ours in that it was again a free reaction time choice task. Endpoint accuracy was also recorded in a recent study (Boyd et al., 2009) investigating neural correlates of movement planning in a reaching task with restricted and unrestricted planning. The authors found that endpoint accuracy was reduced in the restricted planning condition. In their study a single target had to be reached after a go signal that followed a restricted (100 ms) or unrestricted (1,000 ms) stimulus presentation interval. However, neither movement time nor reaction time were controlled, only response times from go signal to movement end were measured. Response time could vary within a 4 s window and was elevated for the restricted planning condition. Reaction time was not controlled because movement could be initiated any time after the go cue. During the unrestricted planning condition the authors found additional brain activity in the medial frontal gyrus, pre-SMA, putamen and cerebellum.

While there have been a number of studies investigating the effect of neuromuscular noise on motor control (Harris and Wolpert, 1998; Faisal et al., 2008; Selen et al., 2009), studies that investigate the noise contribution of the central nervous system are rare. The neurophysiological basis of limited motor planning on endpoint variability has been previously investigated in monkeys by Churchland et al. (2006a) who found that variation in perparatory neural activity approximately predicts half of the variability in peak velocity of the movement. In a second study Churchland et al. (2006b) also report that changes in firing rate variability in premotor cortex are an indication of planning progress during motor preparation. In particular, the firing rate variability was found to be initially high and to decline after target onset and go cue. Longer reation times were found to occur when firing rates had higher variability. These results are consistent with the notion that shorter motor preparation times lead to higher movement variability, which is exactly what we observed in our experiment.

Although there have been attempts in applying computational models of noisy decision-making to *continuous* motor planning (Cisek et al., 2009; Resulaj et al., 2009; Thura et al., 2012; Ramakrishnan and Murthy, 2013), typically noisy planning models are perceptual evidence accumulation models for *discrete* decision-making (Laming, 1968; Ratcliff, 1978, 1988; Carpenter, 1981; Townsend and Ashby, 1983; Luce, 1986; Schweickert, 1993; Ratcliff and L Smith, 2004; Ratcliff and Starns, 2013), very often with just two alternatives. In these models, noisy sensory evidence is accumulated for different response alternatives, where each alternative is typically represented by its own accumulator, such that the number of accumulators directly corresponds to the number of response alternatives. Evidence is accumulated until one of the accumulators reaches a threshold, which corresponds to a decision for the pertinent response alternative. The decision is then executed by a movement. However, the movement itself is usually not considered part of the planning or decision-making process. Such accumulator models for discrete choice can be shown to approximately implement Bayes-optimal decision-making (Laming, 2001; Brown et al., 2009; Bitzer et al., 2015; Noorani and Carpenter, 2016) and can be related to sequential sampling methods (Stone, 1960; Laming, 1968; Link and Heath, 1975; Draglia et al., 1999; Dragalin et al., 2000). Such sampling-based decision-making processes model the transition from prior to posterior through a sequence of small steps. In most models the trade-off between accuracy and the number of steps is determined by a fixed decision boundary, but dynamic decision boundaries have also been studied in models that consider explicit costs for evidence accumulation (Frazier and Yu, 2007; Drugowitsch et al., 2012). The speed-accuracy trade-off can be considered as a special manifestation of the abstract utility-information trade-off when assuming a particular dynamic realization of the prior-posterior transition. Other sampling models of optimal decision-making with limited resources include Markov chain Monte Carlo (MCMC) models, where decisions are made subject to the constraint that only a certain number of samples can be evaluated during the deliberation phase (Vul et al., 2014). Such sampling-based decision-making models can be considered as a particular implementation of information-theoretic bounded rational decision-making when they converge to the appropriate equilibrium distributions (Ortega et al., 2014; Hihn et al., 2018).

From the large family of sampling-based decision-making models, in particular drift diffusion evidence accumulation models have been successfully linked to neurophysiological recordings, especially in the parietal cortex (Wang and Sandholm, 2002; Huk and Shadlen, 2005; Gold and Shadlen, 2007; Yang and Shadlen, 2007; Hanks et al., 2011), as the input to the accumulators is assumed to be Brownian motion where the signal is encoded in the drift. Mainly, four different kinds of drift diffusion models are distinguished: race models (Vickers, 1970, 1979; Busemeyer and Diederich, 2002; Bogacz et al., 2006; Bitzer et al., 2014; Insabato et al., 2014), mutual inhibition models (Usher and Mcclelland, 2001; Wong et al., 2007), feed-forward inhibition models (Shadlen and Newsome, 2001; Mazurek et al., 2003) and pooled inhibition models (Wang and Sandholm, 2002; Wong and Wang, 2006). In race models accumulators are independent and the first accumulator to reach the threshold wins, whereas in the other models accumulators compete by inhibiting each other. Recently, there has been considerable interest to relate such models to information-theoretic quantities similar to Hick's law, which is often used as a robust empirical finding to validate these models (Usher and Mcclelland, 2001; Usher et al., 2002; McMillen and Holmes, 2006; Leite and Ratcliff, 2010; Hawkins et al., 2012). In particular, Usher and Mcclelland (2001) have studied several kinds of drift diffusion models and found simulated reaction times to vary linearly with the number of alternatives, as required by Hick's law.

Decision-making models that take the cost of computation into account have also been explored in the reinforcement learning literature (Keramati et al., 2011, 2016; Pezzulo et al., 2013; Viejo et al., 2015). In these studies there is typically a trade-off between cheap model-free learning that simply summarizes past experiences and computationally expensive model-based learning that allows for better decision-making at the cost of deeper planning requiring cognitive effort. The choice between the two processes is then mediated by a meta-decision that may take into account the estimated value of additional deliberation, opportunity costs, the uncertainty of the value estimates, and other factors. Compared to our framework, these models are much more specific, making concrete assumptions about how behavior changes dynamically across time depending on the available resources (for example, the number of available planning steps), and where the meta-decision determines the optimal resource level given these constraints. The advantage of such concrete models (just like their neurally inspired cousins from the previous paragraph) is that they also allow to study adaptation and sequential decision-making effects. In contrast, the framework of information-theoretic bounded rationality operates on a more abstract level that ignores concrete mechanisms of decision-making, but just considers the basic trade-off between utility and information, answering the question of what is the highest achievable utility with a given amount of information (i.e., a given amount of states that can effectively be discriminated). Such an abstraction has both advantages and disadvantages. The disadvantage is clearly that predictions regarding the precise dynamics and adaptation of the decision-making process are limited, unless more constraints are considered in the optimization problem. The advantage is that the framework can be applied more generally and normatively, both to biological information processing and machines. As a consequence, rationality theory with computational constraints may provide a bridge between neuroscience and artificial intelligence and bring these two fields closer together again (Gershman et al., 2015; Jordan and Mitchell, 2015; Parkes and Wellman, 2015).

## Author Contributions

SS, SG, and DB designed the experiment. SS performed experiments and analyzed the data. SS and SG generated predictions from computer simulations. SG and DB supervised the project. SS and DB wrote the paper.

### Conflict of Interest Statement

The authors declare that the research was conducted in the absence of any commercial or financial relationships that could be construed as a potential conflict of interest.
