# Supplementary material for: Quantifying Motor Task Performance by Bounded Rational Decision Theory
Source: Front Neurosci. 2018 Dec 14;12:932. doi: 10.3389/fnins.2018.00932 (PMC6302104; doi:10.3389/fnins.2018.00932)
Supplement: Supplementary file 1 [file Data_Sheet_1.pdf]

frontiers

Supplementary Material: Quantifying motor task performance by bounded rational decision theory

1 DERIVATION OF EQS. (5) AND (6)

In the following, we assume the notation and definitions from Section 2.1 of the main text. All optimizations are over the set of probability densities on $\mathcal{A} \subset \mathbb{R}$.

Lemma S1. *For any* $\beta > 0$ *and fixed prior density* p_0*, the optimization problem*

$$\max_{p(a|w)} \left[\mathbb{E}_{\rho(w)} \left[\mathbb{E}_{p(a|w)} \left[V(a,w) \right] \right] - \tfrac{1}{\beta} \, \mathbb{E}_{\rho(w)} \left[D_{\mathrm{KL}} \left(p(a|w) \| p_0(a) \right) \right] \right] \tag{S1}$$

is equivalent to the constrained optimization problem

$$\max_{p(a|w)} \mathbb{E}_{\rho(w)} \left[\mathbb{E}_{p(a|w)} \left[V(a,w) \right] \right] \quad \textit{s.t.} \quad \mathbb{E}_{\rho(w)} \left[D_{\mathrm{KL}} \left(p(a|w) \| p_0(a) \right) \right] \leq C_\beta \,, \tag{S2}$$

where $C_\beta := \mathbb{E}_{\rho(w)} \left[D_{\mathrm{KL}} \left(p^*_\beta(a|w) \| p_0(a) \right) \right]$*, and* p^*_β *denotes the probability density that maximizes* (S1)*, more precisely,*

$$p^*_\beta(a|w) \ = \ \frac{1}{Z(w)} \, p_0(a) \, e^{\beta V(a,w)} \tag{S3}$$

with normalization constant $Z(w) := \int_{\mathcal{A}} p_0(a) \, e^{\beta V(a,w)} \, da$*.*

Proof. The optimization problem (S1) is the variational formulation of (S2) with Lagrange multiplier β^{-1} (see for example Everett, 1963[1]). For the proof of (S3), we first show that the functional J given by

$$J[p] := \sum_{w \in \mathcal{W}} \rho(w) \left[\mathbb{E}_{p(a|w)}[V(a,w)] - \tfrac{1}{\beta} D_{\mathrm{KL}}(p(a|w) \| p_0(a)) + \lambda \, \mathbb{E}_{p(a|w)}[1] \right] \tag{S4}$$

is strictly concave and therefore has a unique maximum. Note that the last term in J has been added for the extra constraint that $p(a|w)$ must be normalized. Since the expectations of V and 1 are linear in $p(a|w)$, the strict concavity of J follows from the strict convexity of D_{KL} in the first argument, i.e.

$$D_{\mathrm{KL}}(tp_1 + (1-t)p_2 \| q) < t D_{\mathrm{KL}}(p_1 \| q) + (1-t) D_{\mathrm{KL}}(p_2 \| q) \,, \tag{S5}$$

whenever $p_1 \neq p_2$, $p_i \neq q$ for $i = 1, 2$ almost everywhere (outside of a set of measure 0), and $0 < t < 1$. Inequality (S5) follows from the non-negativity of the Kullback-Leibler divergence as follows: For $p_1 \neq p_2$,

[1] Hugh Everett, Generalized Lagrange Multiplier Method for Solving Problems of Optimum Allocation of Resources, Operations Research, Vol. 11, No. 3 (1963), pp. 399-417.

$p_i \neq q$ for $i = 1, 2$ almost everywhere, and $0 < t < 1$, we have

$$
\begin{aligned}
& D_{\mathrm{KL}}(tp_1 + (1-t)p_2 \| q) - tD_{\mathrm{KL}}(p_1\|q) + (1-t)D_{\mathrm{KL}}(p_2\|q) \\
& = t \int_{\mathcal{A}} p_1(a) \log \frac{tp_1(a) + (1-t)p_2(a)}{q(a)}\, da + (1-t) \int_{\mathcal{A}} p_2(a) \log \frac{tp_1(a) + (1-t)p_2(a)}{q(a)}\, da \\
& \quad -t \int_{\mathcal{A}} p_1(a) \log \frac{p_1(a)}{q(a)}\, da - (1-t) \int_{\mathcal{A}} p_2(a) \log \frac{p_2(a)}{q(a)}\, da \\
& = t \int_{\mathcal{A}} p_1(a) \log \frac{tp_1(a) + (1-t)p_2(a)}{p_1(a)}\, da + (1-t) \int_{\mathcal{A}} p_2(a) \log \frac{tp_1(a) + (1-t)p_2(a)}{p_2(a)}\, da \\
& = -tD_{\mathrm{KL}}(p_1 \| tp_1 + (1-t)p_2) - (1-t) D_{\mathrm{KL}}(p_2 \| tp_1 + (1-t)p_2) \\
& < 0\,,
\end{aligned}
$$

where we have used that $D_{\mathrm{KL}}(p\|p_0) \geqslant 0$ with equality if and only if $p = p_0$ almost everywhere. This proves (S5). In particular, J has a unique maximum p^*, which satisfies

$$
\frac{\delta J}{\delta p}(a, w)\bigg|_{p=p^*} = 0 \tag{S6}
$$

for almost every $a \in \mathcal{A}$ and $w \in \mathcal{W}$. Since

$$
\frac{\delta J}{\delta p}(a, w) = V(a, w) - \frac{1}{\beta} \log \frac{p(a|w)}{p_0(a)} - \frac{1}{\beta} + \lambda\,,
$$

we obtain from (S6)

$$
p(a|w) = p_0(a)\, e^{\beta V(a,w) - 1 + \beta\lambda}\,,
$$

where $\lambda \in \mathbb{R}$ must be chosen such that $\int_{\mathcal{A}} p(a|w) da = 1$, i.e. $e^{1-\beta\lambda} = \int_{\mathcal{A}} p_0(a)\, e^{\beta V(a,w)}\, da =: Z(w)$, which proves (S3). This concludes the proof of Lemma S1.

Lemma S2. *The optimization problem*

$$
\max_{p_0(a), p(a|w)} \Big[\mathbb{E}_{\rho(w)} \left[\mathbb{E}_{p(a|w)} \left[V(a, w) \right] \right] - \tfrac{1}{\beta}\, \mathbb{E}_{\rho(w)} \left[D_{\mathrm{KL}} \left(p(a|w) \| p_0(a) \right) \right] \Big] \tag{S7}
$$

is equivalent to

$$
\max_{p(a|w)} \Big[\mathbb{E}_{\rho(w)} \left[\mathbb{E}_{p(a|w)} \left[V(a, w) \right] \right] - \tfrac{1}{\beta}\, I(W; A) \Big] \tag{S8}
$$

where $I(W;A)$ denotes the mutual information between the random variables W and A with joint distribution $p(a, w) := \rho(w)p(a|w)$. Moreover, the unique solution can be found by a Blahut-Arimoto-type algorithm, which consists of iterating the equations

$$
p(a|w) = \frac{1}{Z(w)}\, p(a)\, e^{\beta V(a,w)} \tag{S9}
$$

$$
p(a) = \sum_{w \in \mathcal{W}} \rho(w) p(a|w) \tag{S10}
$$

until convergence is achieved.

Proof. Optimizing for the prior p_0 in (S7) has a unique solution p_0^*, since the Kullback-Leibler divergence is also strictly convex in the second entry due to the strict concavity of the logarithm. Similarly as in the proof of Lemma S1, setting the functional derivative of J in (S4) with respect to p_0 to zero, where the normalization term for the posterior is replaced by a normalization term for the prior p_0, i.e. $\lambda \mathbb{E}_{p_0}[1]$, yields

$$\sum_w \rho(w) \frac{1}{\beta} \frac{p(a|w)}{p_0(a)} + \lambda = 0$$

which shows that $p_0^*(a) = \sum_w \rho(w) p(a|w)$, i.e. the optimal prior is the marginal of the posterior. Hence,

$$\mathbb{E}_{\rho(w)}\big[D_{\mathrm{KL}}(p(a|w)\|p_0^*(a))\big] = \sum_{w\in\mathcal{W}} \int_{\mathcal{A}} p(a,w) \log \frac{p(a,w)}{p(a)\rho(w)}\, da = I(W;A)$$

where $p(a,w) := \rho(w)p(a|w)$. This proves the equivalence of (S7) and (S8). Finally, note that (S8) is equivalent to the classic rate distortion problem with $-V$ as distortion function, for which the convergence of the alternating Blahut-Arimoto optimization algorithm given by Eqs. (S9)-(S10) has been proved for example in Csiszár, 1975[2]. This concludes the proof of Lemma S2.

[2] I. Csiszár, I-divergence geometry of probability distributions and minimization problems, The Annals of Probability, Vol. 3, No. 1 (1975), pp. 146-158.

2 SUPPLEMENTARY TABLES

TABLE S1 | Average information in bit for each subject and each condition. S1−S10 index the ten different subjects. RT_1 is the fast reaction time condition, RT_2 is the slow reaction time condition. The target probabilities were uniform (ρ_u) or non-uniform (ρ_{nu}).

	S 1		S 2		S 3		S 4		S 5	
	RT_1	RT_2	RT_1	RT_2	RT_1	RT_2	RT_1	RT_2	RT_1	RT_2
ρ_u	1.641	1.494	1.657	1.697	1.458	1.555	1.328	1.431	1.640	1.570
ρ_{nu}	1.169	1.148	0.954	1.043	0.999	1.104	1.062	1.075	1.147	1.112

	S 6		S 7		S 8		S 9		S 10	
	RT_1	RT_2	RT_1	RT_2	RT_1	RT_2	RT_1	RT_2	RT_1	RT_2
ρ_u	1.163	1.442	1.218	1.520	1.246	1.729	1.444	1.674	1.388	1.529
ρ_{nu}	0.742	0.998	1.024	0.983	0.881	1.147	1.003	1.102	1.125	1.165

TABLE S2 | Average utility for each subject and each condition. Utility was binary depending on whether the angular coordinate of the movement endpoint aligned with the angular coordinate interval of the target. S1−S10 index the ten different subjects. RT_1 is the fast reaction time condition, RT_2 is the slow reaction time condition. The target probabilities were uniform (ρ_u) or non-uniform (ρ_{nu}).

	S 1		S 2		S 3		S 4		S 5	
	RT_1	RT_2	RT_1	RT_2	RT_1	RT_2	RT_1	RT_2	RT_1	RT_2
ρ_u	0.945	0.939	0.976	0.975	0.939	0.965	0.913	0.941	0.939	0.959
ρ_{nu}	0.954	0.957	0.854	0.927	0.917	0.961	0.954	0.960	0.952	0.958

	S 6		S 7		S 8		S 9		S 10	
	RT_1	RT_2	RT_1	RT_2	RT_1	RT_2	RT_1	RT_2	RT_1	RT_2
ρ_u	0.874	0.948	0.890	0.959	0.852	0.980	0.912	0.970	0.898	0.939
ρ_{nu}	0.722	0.920	0.954	0.937	0.885	0.957	0.922	0.960	0.937	0.957

3 SUPPLEMENTARY FIGURES

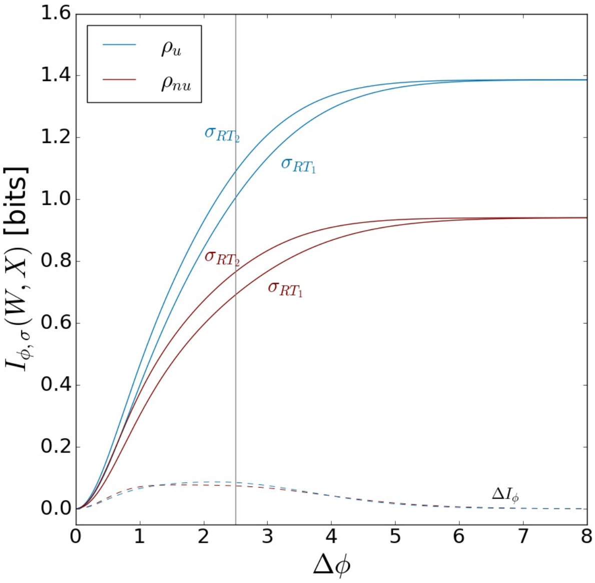

FIGURE S1 | Specification of the angular target distance $\Delta\phi$ in the experimental design. For the fast and slow reaction time condition RT_1 and RT_2 subjects perform with different endpoint variability σ_{RT_1} and σ_{RT_2}. Here we present an example based on measured endpoint variability of subject S4. This variability is mapped into a mutual information $I_{\phi,\sigma}(W,X)$ that quantifies how well targets w can be told apart when just looking at the movement endpoints x belonging to each target. Naturally, this depends on the distance $\Delta\phi$ between targets. If targets are in exactly the same location $(\Delta\phi = 0)$ they cannot be distinguished $(I_{\phi,\sigma} = 0)$. If targets are very far apart ($\Delta\phi \gg \sigma$), they can be told apart perfectly ($I_{\phi,\sigma} = I_{\max}$) and movement variability is irrelevant. For intermediate distance $\Delta\phi$ the difference ΔI_ϕ in mutual information for the two reaction time conditions is non-zero, because the target distance is on the same order of magnitude as σ. We set an angular distance of $\Delta\phi = 2.5$, because this belongs to a range of $\Delta\phi$-values where ΔI_ϕ for different reaction times is most sensitive.

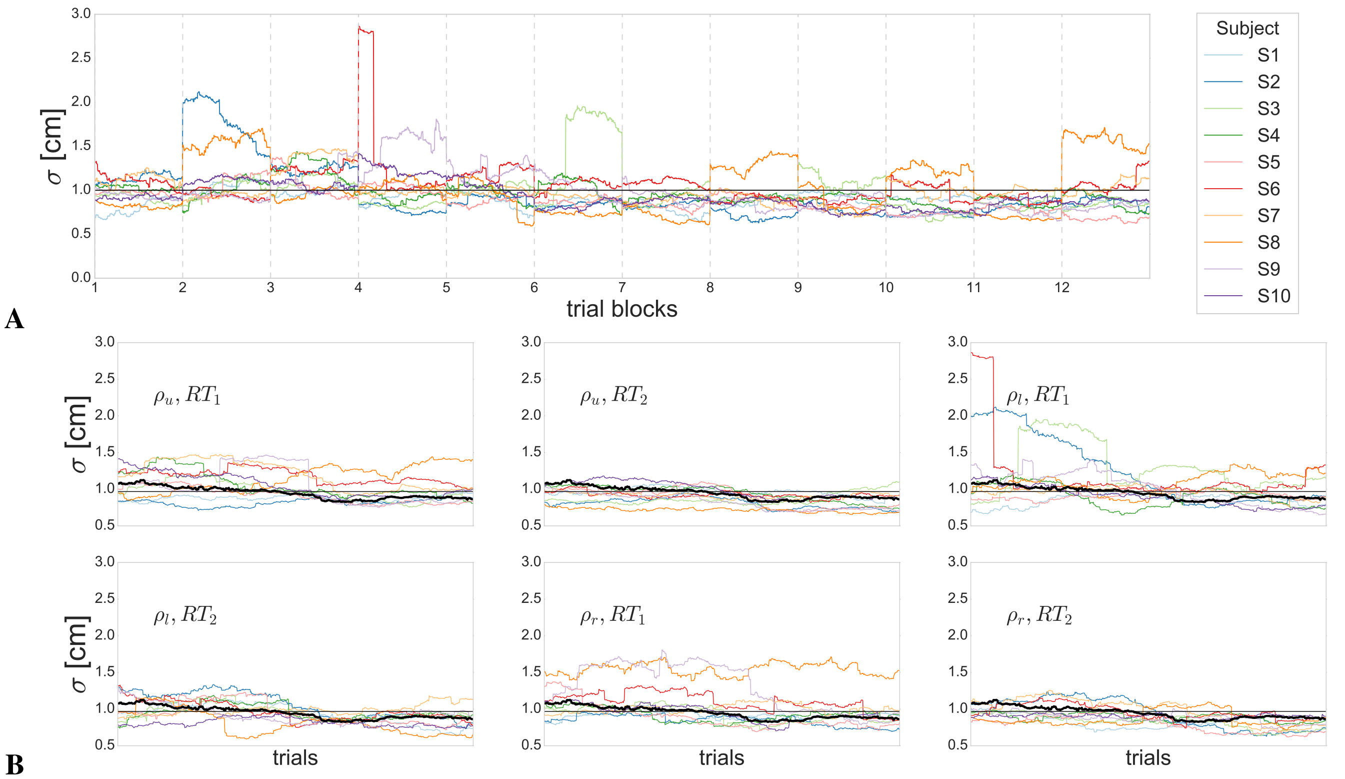

FIGURE S2 | Endpoint variance across experimental blocks. The recorded trial sequences were analyzed for each subject individually. The standard deviation σ is computed for all trial blocks with a sliding window of 200 trials. **(A)** Over the course of the experiment endpoint variances slightly decreased and indicate that subjects were still improving their task performance due to learning. **(B)** Within blocks of particular conditions (defined by reaction time and world state distribution), endpoint variance remains relatively stable and suggests no systematic effects of fatigue. The median over subjects is shown in black for trial blocks of all six experimental conditions.

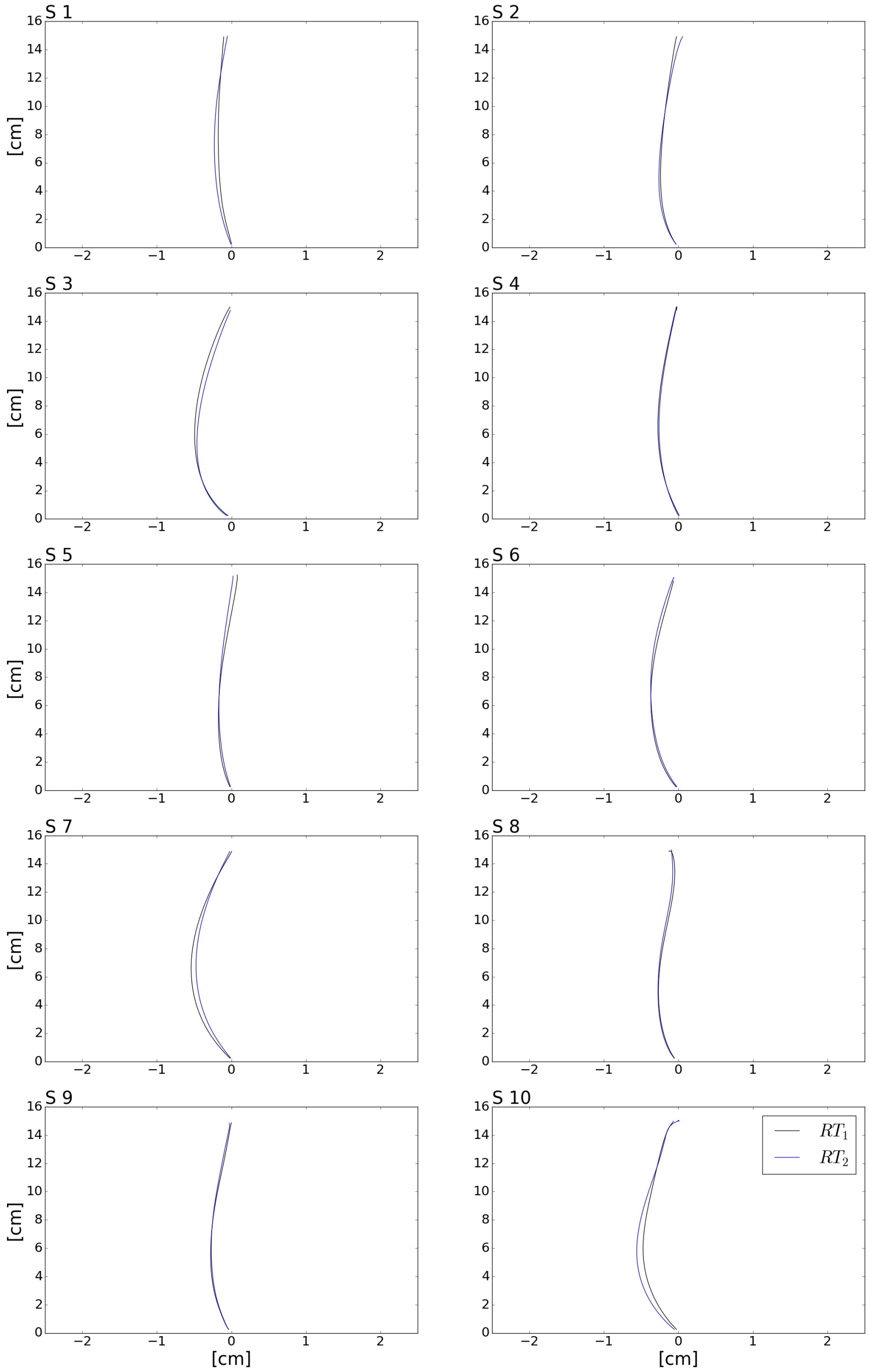

FIGURE S3 | Mean movement trajectories for the two different reaction time conditions for each subject under uniform target distribution. Trajectory path towards uniformly distributed targets were rotated by target angles and averaged for fast (RT_1) and slow (RT_2) reaction times. Trajectories have similar shape for individual subjects. There is no systematic difference between movement paths in the two reaction time conditions.

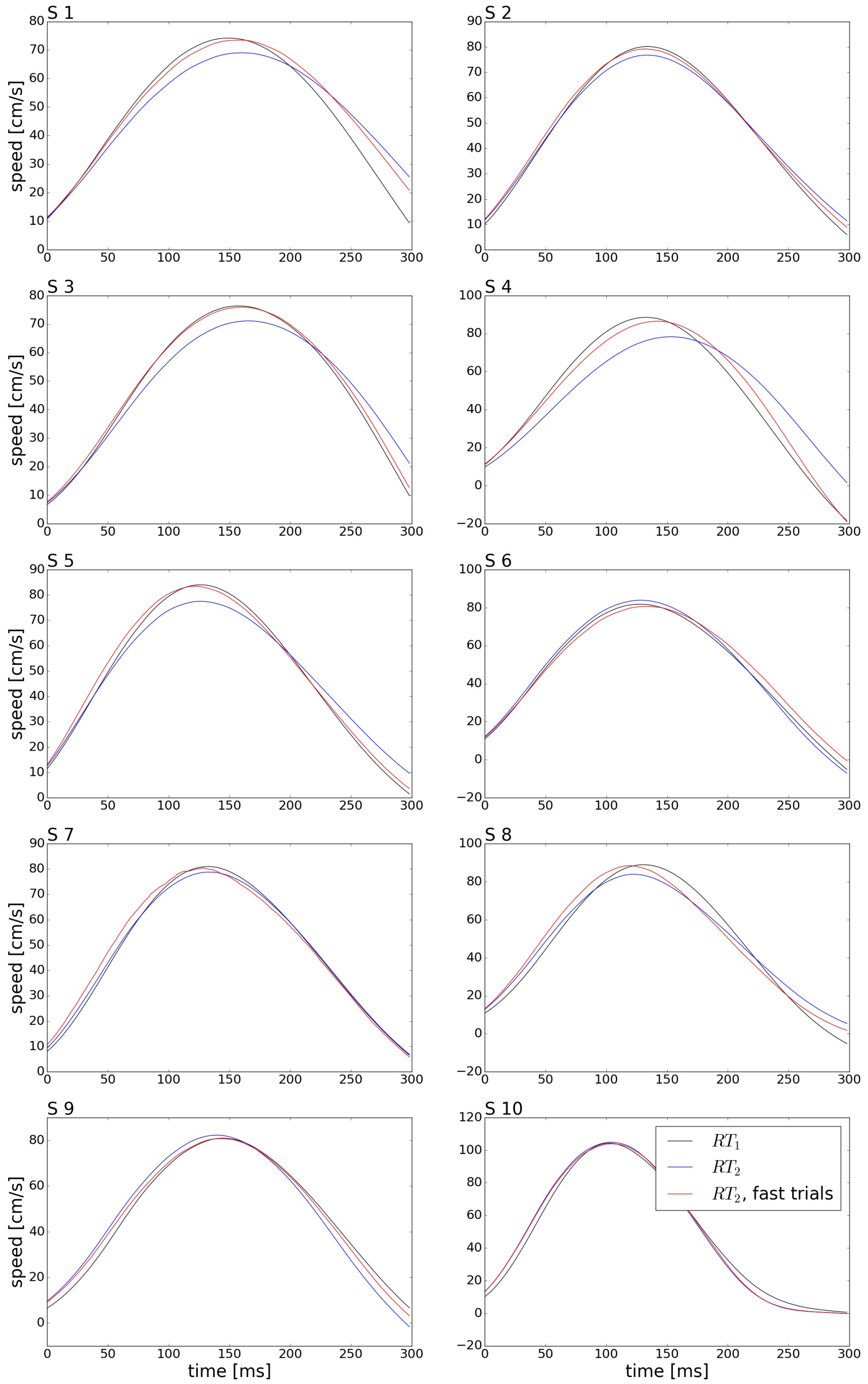

FIGURE S4 | Mean movement speeds for the two different reaction time conditions for each subject under uniform target distribution. Subjects' movement speed during movement execution were averaged for fast (RT_1) and slow (RT_2) reaction times. Speed profiles have their maximum midway. Movements performed under the fast reaction time condition have on average a slightly higher maximum speed. To control for the effect of movement speed during execution on endpoint variance, we selected a "high-speed" subset of trajectories in the slow reaction time condition that match on average the speed of the fast reaction time condition. The mean speed profiles of this subset are shown in red.

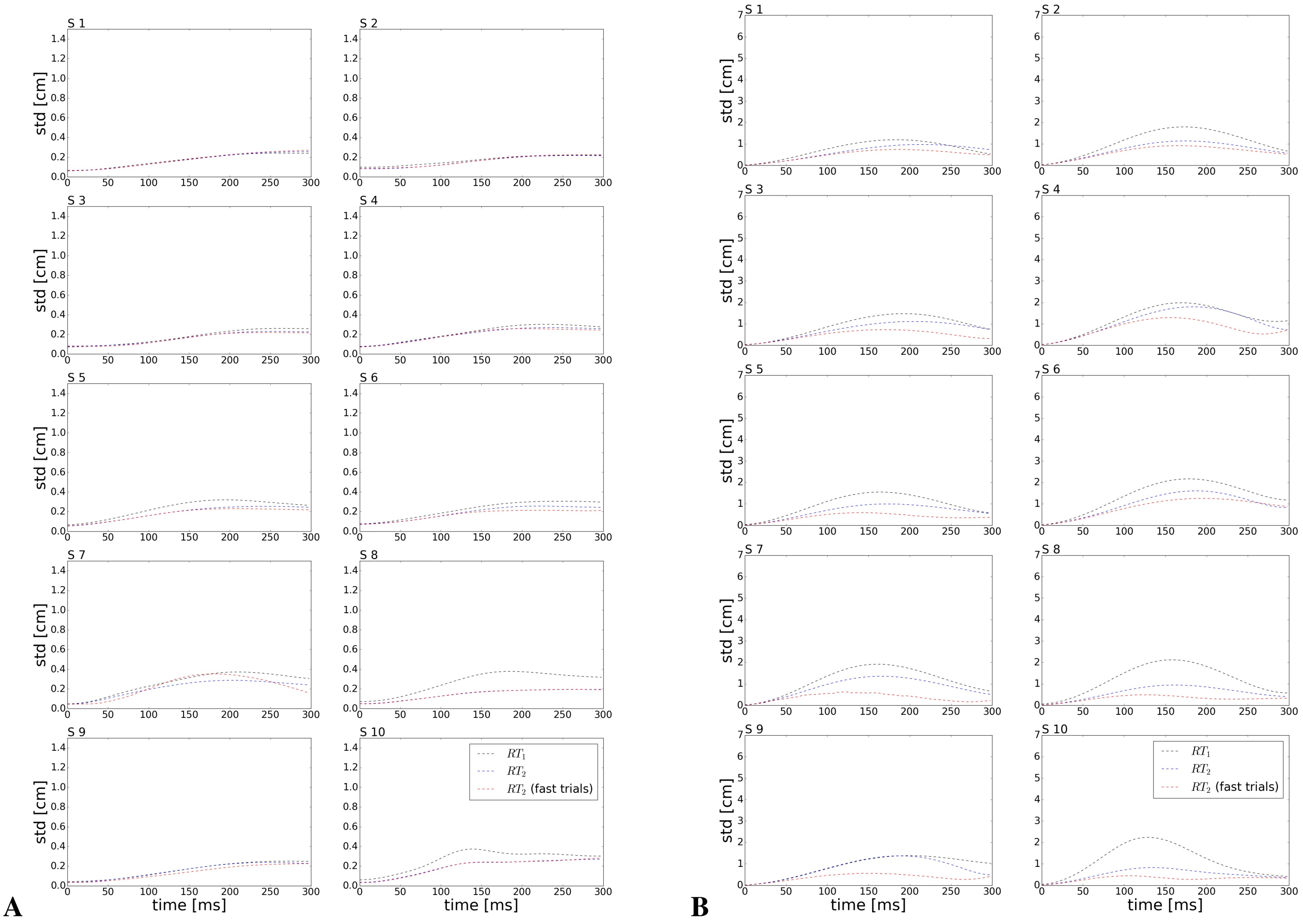

FIGURE S5 | Standard deviation of movement trajectories for the two different reaction time conditions for each subject under uniform target distribution. In the fast reaction time condition RT_1, variance is systematically higher than in the slow reaction time condition RT_2. This difference remains when considering a subset of trajectories in RT_2 that is matched for speed and marked in red in the figure–compare Supplementary Figure S4. The increase in movement variability can therefore not be explained by the small difference in peak movement speed, but must be related to the shortened planning phase. Panel **(A)** shows standard deviations in the x-dimension, panel **(B)** in the y-dimension.

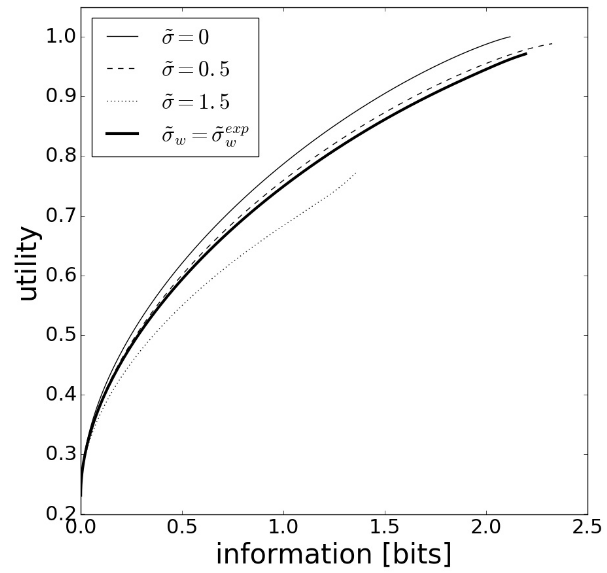

FIGURE S6 | Motor execution noise. Different efficiency frontiers arise for different levels of motor execution noise. In the absence of execution noise ($\tilde{\sigma} = 0$), motor planning can precisely determine the endpoint of the movement. In this case the expected utility will be the highest (compared to $\tilde{\sigma} > 0$) and information just measures uncertainty arising from the imperfect planning process. For higher levels of execution noise, the efficiency frontier moves downward on the utility axis, including the maximal achievable utility. In this case information comprises uncertainty arising both from planning and execution processes. Here, $\tilde{\sigma}_w^{exp}$ denotes the experimentally determined target-specific movement noise of a particular subject and illustrates the pertinent efficiency frontier.

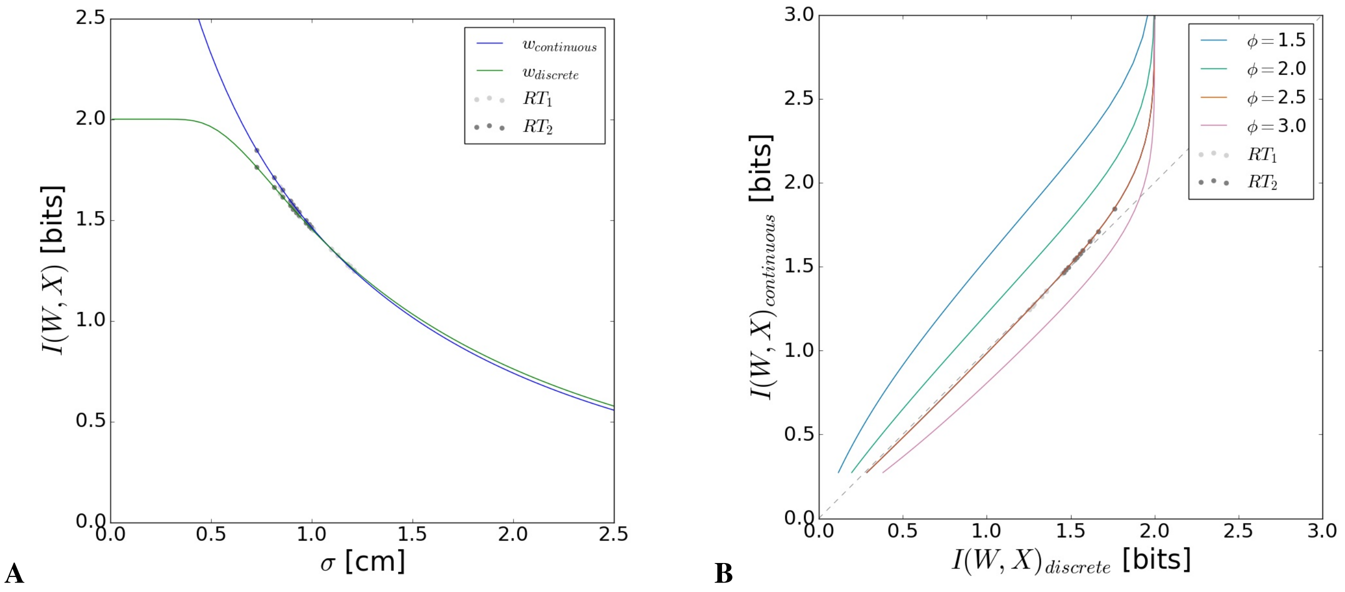

FIGURE S7 | Comparison of discrete and continuous mutual information. Continuous information is computed for an interval $[w_{\min}, w_{\max}] = [-4.7°, +4.7°]$. Discrete mutual information is computed for the four targets in our experiment. Panel **(A)** shows mutual information $I(W;X)$ as a function of endpoint spread σ. For σ-values occuring in the experiment the two curves are very similar, for small σ there is a deviation. In the continuous case with arbitrarily small σ, arbitrarily large information values can be achieved, as arbitrarily many targets can be distinguished. In the discrete case mutual information levels off as there are only finitely many targets that can be distinguished. Panel **(B)** plots discrete and continuous mutual information against each other for different target distances $\Delta\phi$. For the target distance $\Delta\phi = 2.5$ and the experimentally observed variances, continuous and discrete information are virtually identical. Therefore, we can consider our discrete setup as a valid approximation of the continuous case.
